# Deep mutational scanning of *S. pyogenes* Cas9 reveals important functional domains

**DOI:** 10.1038/s41598-017-17081-y

**Published:** 2017-12-04

**Authors:** Jeffrey M. Spencer, Xiaoliu Zhang

**Affiliations:** 1 Department of Biology and Biochemistry, University of Houston, Houston, Texas 77204 USA; 2 Center for Nuclear Receptors and Cell Signaling, University of Houston, Houston, Texas 77204 USA

## Abstract

RNA-guided endonucleases (RGENs) have invigorated the field of site-specific nucleases. The success of *Streptococcus pyogenes* Cas9 (SpCas9) has led to the discovery of several other CRISPR-associated RGENs. As more RGENs become available, it will be necessary to refine their activity before they can be translated into the clinic. With this in mind, we sought to demonstrate how deep mutational scanning (DMS) could provide details about important functional regions in SpCas9 and speed engineering efforts. Consequently, we developed a nuclease screening platform which could distinguish active Cas9 mutants. We screened a library of 1.9 × 10^7^ with over 8500 possible non-synonymous mutations and inferred the effects of each mutation using DMS. We demonstrate that the RuvC and HNH domains are the least tolerant regions to mutation. In contrast, the Rec2 and PI domains tolerate mutation better than other regions. The mutation information defined in this work provides a foundation for further SpCas9 engineering. Together, our results demonstrate how DMS can be a powerful tool to uncover features important to RGEN function. Application of this approach to emerging RGENs should enhance their engineering and optimization for therapeutic and other applications.

## Introduction

RNA-guided endonucleases (RGENs) adapted from bacterial CRISPR (Clustered Regularly Interspaced Short Palindromic Repeats) immunity have rapidly advanced the field of genetic engineering^1^. The ease of retargeting these proteins to distinct sites using simplistic base-pairing rules has driven the widespread adoption of gene-editing techniques into many laboratories and facilitated the transition of these tools into promising applications^2^. Of these, *Streptococcus pyogenes* Cas9 (SpCas9) was the first system to be exploited as a gene editing tool and remains the most prominent RGEN^3,4^.

Despite its advantages, inherent properties of the SpCas9 system limit its potential. Shortly after its implementation, studies raised concerns about the specificity of the enzyme which could hinder its therapeutic application^5,6^. In addition, the requirement of the Protospacer Adjacent Motif (PAM) limits the number of sites which can be targeted. Moreover, varying guide-RNA (gRNA) activities necessitate functional characterization of gRNAs prior to application, hence complicating gRNA choice^7,8^. Finally, the bacterial origin of the SpCas9 protein contributes to the elicited specific immune response when delivered by adeno-associated virus in mice, making immunogenic side effects a concern^9^. Combined, these properties present challenges to SpCas9 realizing the ambitious goals of many gene editing applications.

Several different strategies have been used to address some of these challenges. Specifically, protein engineering efforts have uncovered mutations in the protein which alter its PAM recognition^10^ and enhance its fidelity^11,12^. Further modifications to the protein and gRNA design, such as FokI fusions, paired nicking, and truncated gRNAs, have provided additional improvements to specificity^13,14^. However, each of these strategies imposes a unique set of restrictions, adding to the complexity of target design. Others have focused efforts on identifying novel SpCas9 orthologs^15–17^ or proteins from distinct CRISPR classes^18–20^, generating a gene editing toolbox which may overcome as a suite what cannot be accomplished by any protein alone. To accomplish this goal, each of these proteins must be characterized and optimized before they can be applied to sensitive therapeutic applications.

Optimization of RGENs can be time consuming. With this in mind, we sought to demonstrate how Deep Mutational Scanning (DMS) can be used to accelerate RGEN protein engineering by uncovering features amenable to mutation^21^. To this end, we generated a bacterial selection system to assay SpCas9 nuclease function. We employed this nuclease screen to gather information about amino acids important to SpCas9’s ability to cleave DNA. As a result, we identified several amino acid mutations which impact specificity and activity of this RGEN. Together, these mutations and the domains in which they reside outline a path for further modification and highlight the utility of this approach for optimizing the expanding toolbox of RGENs.

## Materials and Methods

### Selection Plasmid Cloning

The positive selection plasmid, pACYC184 -tacP- ccdB -T2 (Supplementary Fig. 1), was generated from a modified pACYC184 plasmid (NEB) in which URA3 had been inserted under the control of the J23119 promoter using SacII. A T2 target site (GACCCCCTCCACCCCGCCTCCGG) was inserted into this plasmid at the AvaI site using annealed oligos. Another four copies of the T2 target site were cloned as oligos into the SalI site, simultaneously disrupting the tetracycline resistance gene. The tac promoter (P_tac_) was added to the LacZ-ccdB fusion expression cassette by PCR. Briefly, the LacZ-ccdB cassette was amplified from pCRII (Invitrogen) using tacP For and ccdB Rev and cloned into the AflII digested, Klenow fragment (NEB) treated pACYC184 –URA3 –T2 vector, removing the majority of the URA3 coding sequence in the process and generating pACYC184 -tacP- ccdB -T2.

The negative selection plasmid, pH3 – OT9 (Supplementary Fig. 2), was created from a modified pH3U3-mcs (a gift from Scot Wolfe, Addgene plasmid # 12609). pH3 was generated by digesting pH3U3-mcs with BamHI and XhoI followed by Klenow fragment treatment and self-ligation. pH3 was subsequently digested with AgeI and AatII to ligate oligos consisting of 2 copies of the off-target site (5′-GCCCCCACCCACCCCGCCTCCGG-3′).

The SpCas9 expression plasmid, pUC-ProD-Cas9-T2 (Supplementary Fig. 3), was assembled from a pUC backbone vector to incorporate the ProD insulated promoter^22^ which was synthesized as a gBlock (IDT). The J23119 promoter, T2 targeting gRNA, and terminators were inserted from synthesized oligos. The cloning vector pUC-ProD-LacZ-T2 (Supplementary Fig. 4) was generated by cloning a LacZ amplicon between two Esp3I sites, to allow for golden gate assembly. Primers, plasmids, and oligos used for cloning can be found in Supplementary Tables 1, 2, and 3 respectively.

### Bacterial Selection Assays

For positive selection, US0hisB-pyrF-, a gift from Scot Wolfe (Addgene plasmid # 12614), harboring the plasmid pACYC184 -tacP- ccdB -T2 (Supplementary Fig. 1), which contains a chloramphenicol resistance gene, were used to perform positive selections for SpCas9 variants. To generate competent cells used for these assays, cells were transformed with the selection plasmid and then cultured with tetracycline (10 µg/mL, Sigma) and chloramphenicol (33 µg/mL, Sigma) to generate electrocompetent cells as previously described^23^. SpCas9/gRNA expression plasmids (20 ng) were added to 20 µl of competent cells and pulsed at 1800V. Cells were recovered in 1 mL SOC media for 30 minutes at 37 °C with shaking prior to plating serial dilutions on selection (tetracycline 10 µg/mL, ampicillin 100 µg/mL (Sigma), 1 mM IPTG (Sigma)) and control plates (tetracycline 10 µg/mL, ampicillin 100 µg/ml). Plates were incubated at 37 °C overnight before colonies were counted at the lowest dilution in which colonies could be distinguished. Survival was calculated as a ratio of colonies able to survive on selection plates as compared to control plates.

For negative selection, DH5α cells harboring pH3 – OT9 (Supplementary Fig. 2), containing a kanamycin resistance gene, were used to perform negative selection of SpCas9 variants. Electrocompetent cells were generated using the same technique as described for the positive selection cells, replacing the antibiotics with kanamycin (50 µg/mL, Sigma). Cells were electroporated as above and plated on selection plates (kanamycin 50 µg/mL, ampicillin 100 µg/mL) and control plates (ampicillin 100 µg/mL).

### SpCas9 Mutant Library Cloning

A template plasmid, EF- RBS-SpCas9 (Supplementary Fig. 5), was created with a ribosome binding site attached to a human codon optimized SpCas9 protein coding sequence (Supplementary Fig. 6) and the appropriate Esp3I sites to be used for error-prone PCR by POE-PCR^24^. Error-prone PCR was performed with the Mutazyme II polymerase (Agilent Technologies) in 1X Mutazyme buffer (Agilent Technologies); 200 ng template was amplified with EF For and SV40 For in a 50 µl reaction with the following cycles: 95 °C for 2 minutes, 20 cycles of 72 °C (−1 °C/cycle) for 30 seconds, 72 °C for 4 minutes and 30 seconds, 15 cycles of 94 °C for 30 seconds, 52 °C for 30 seconds, 72 °C for 4 minutes and 30 seconds, and a final extension at 72 °C for 10 minutes. The resulting amplicons were loaded on a gel and the appropriate amplicon at 4.4 kb was gel extracted and purified with a Qiagen gel extraction column.

Six 20 µl golden gate cloning reactions were set up with 75 ng PCR product and 50 ng pUC-ProD-LacZ-T2 (Supplementary Fig. 4) with 10 U Esp3I (Thermo Fisher Scientific) and 400 U T4 DNA Ligase (NEB) in 1X Ligase Buffer (NEB). The ligation reactions were put in a thermocycler running the program: 60 cycles of 37 °C for 5 minutes, 16 °C for 5 minutes, a final digestion at 37 °C for 30 minutes, and heat inactivation at 65 °C for 20 minutes.

The ligations were ethanol precipitated using standard techniques and resuspended in 9 µl of water. The purified ligations were transformed into five 25 µl aliquots of 10 G Elite electrocompetent cells (Lucigen), pulsed at 1800V, and recovered at 37 °C with shaking in 1 mL Recovery Media (Lucigen) for 1 hour. Upon recovery, the separate transformations were pooled and 20 µl was removed for quantification of total transformants by titration on ampicillin (100 µg/mL), X-gal plates (120 µg/mL). The remaining pool of cells was added to a 125 mL Erlenmeyer flask containing 15 mL LB media supplemented with ampicillin (100 µg/mL) and incubated at 37 °C with shaking for 3 hours. An aliquot of 20 µl was removed from the resulting cell suspension and titrated by plating 5 µL serial dilutions on selection plates (ampicillin 100 µg/mL (Sigma)). Plates were incubated at 37 °C overnight before colonies were counted at the lowest dilution in which colonies could be distinguished. These counts were then extrapolated to determine the expected number of total transformants. The remaining culture was split in half and spun down at 4000 g at 4 °C for 20 minutes, the media was removed, one pellet was resuspended in 15% glycerol LB and frozen, and the other pellet was purified with a Qiagen miniprep kit following the manufacturer’s instructions.

### Library Selection

For each replicate, the library of 1.9 × 10^7^ transformants was transformed into six 50 µl aliquots of negative selection competent cells. The transformations were set up with 120 ng of library DNA in half of the transformations and 250 ng of DNA for the other 3 transformation reactions. Each transformation was recovered at 37 °C for one hour upon addition of 1 mL of SOC media. Following recovery, the transformations were mixed; 50 µl was removed to perform titrations on negative selection and control plates. The remaining transformation mixture was plated in 120 µl aliquots on 46 100 mm × 15 mm negative selection plates and incubated at 37 °C overnight. Each transformation yielded roughly a 3 fold overrepresentation of the total number transformants from the initial library as determined by serial dilutions of a 20 µL aliquot plated on ampicillin (100 µg/mL) in a manner similar to library creation. After overnight incubation, 1 mL of LB (Sigma) supplemented with ampicillin was added to the plates and the colonies were scraped from the plates using a rubber cell scraper. The cell solution was transferred to a 50 mL conical tube and vortexed to mix. The cell suspension was then pelleted at 5000 rpm for 10 minutes. The cell pellet was then resuspended in 10 mL ampicillin LB and distributed into four 500 µl aliquots which were subsequently purified with Qiagen miniprep columns following the manufacturer’s recommendations.

Following DNA purification, 1 µg from each prep (totaling 4 µg) was mixed and incubated in a 40 µl reaction with 40 U of HindIII (NEB) and 40 U of NdeI (NEB) in NEB buffer 2.1 for 1 hour to remove the negative selection plasmid and any residual LacZ plasmids which were still present in the library. This reaction was purified with a Zymoclean spin column (Zymo Research) following the manufacturer’s recommendations and eluted in 10 µl water. The eluted DNA was divided equally into 6 transformation reactions consisting of 50 µl of positive selection cells which were recovered for 30 minutes in 1 mL SOC per reaction. Again, the cells were mixed following recovery and 50 µl was removed to perform titrations on control and positive selection plates. The remaining recovered cells were plated on 46 100 mm × 15 mm positive selection plates and incubated at 37 °C overnight. The transformations resulted in roughly 3 fold overrepresentation of the initial library size based on serial dilutions of a 20 µL aliquot plated on ampicillin (100 µg/mL) in a manner similar to library creation. The cells were harvested in the same manner as the negative selection cells.

### Mammalian Expression gRNA Cloning

Plasmids used to express gRNAs for GFP disruption were cloned into a U6 plasmid, pCRII – U6 gRNA (Supplementary Fig. 7), by digesting with Esp3I and ligating in annealed oligos with the defined protospacer sequences. GFP-targeting gRNAs are based on the gRNAs from^11^. Oligos used to clone each gRNA can be found in Supplementary Table 3.

### Specific SpCas9 Mutant Cloning

Overlap extension PCR was used to generate specific single and combination mutants. Forward and reverse primers were created with the mutations of interest. These primers along with G-Cas For and G-Cas Rev (for mammalian expression, using the bacterial expression plasmid as template to help distinguish mutated products from template) or EF For and SV40 For (for bacterial expression, using the mammalian expression plasmid as template to help distinguish mutated products from template) were used to generate the overlapping fragments necessary to produce the chosen mutation combination. The fused PCR products were either digested with BstXI and BsrGI-HF (NEB) and ligated back into the mammalian expression vector EF-SpCas9 (Supplementary Figure 1) for mammalian expression or used for golden gate assembly into pUC-ProD-LacZ-T2 for bacterial expression as described above. All primers used for mutation PCRs can be found in Supplementary Table 1.

### Cell Culture and Transfections

U2OS cells (ATCC HTB-96) were cultured in DMEM (GE Life Sciences) supplemented with 10% FBS (Fetal Bovine Serum, Gemini Bio-Products) and 1% penicillin/streptomycin (Invitrogen). Cells were grown in incubators at 37 °C in 5% CO_2_ with 95% relative humidity. U2OS-GFP cells were generated by infection with a lentivirus expressing a GFP with a c-terminal tag sequence (HGFPPEVEEQDDGTLPMSCARRAAWTDIKRPRL) under control of the MSCV promoter at a low MOI. A single colony expressing GFP was isolated by limiting dilution and expanded for use in GFP disruption assays.

For GFP disruption assays, U2OS-GFP cells were seeded in 96 well plates 24 hours prior to transfection (1.5 × 10^4^ cells per well). Cells were transfected in duplicate with 0.5 µl of PEI per well and 125 ng plasmid DNA (93 ng SpCas9 expression plasmid, EF-SpCas9 (Supplementary Fig. 8), and 31 ng gRNA expression plasmid (Supplementary Fig. 7)). Control transfections were performed with a plasmid expressing a non-targeting gRNA. Transfection reagent was removed from the cells after 24 hours of exposure. Cells were allowed to grow out for 5 days before being split into 3 wells of a 96 well plate. Ten days after transfection, the cells were pooled together and analyzed by Flow cytometry on a Becton Dickinson FACSAria (BD Biosciences). Cells were gated on untransfected control cells, setting a threshold of 2% GFP negative cells.

For western blots, U2OS-GFP cells were seeded in 24 well plates prior to transfection (8.3 × 10^4^ cells per well). Cells were transfected with 2.76 µl of PEI and 691 ng of SpCas9 expression plasmid DNA per well. Protein was harvested 48 hours post-transfection with 50 µl of RIPA buffer supplemented with protease inhibitor cocktail (Roche). Lysates were spun at 4 °C for 10 minutes at 15000 rpm and the supernatant transferred to fresh tubes. Protein concentrations were quantified with the Bradford assay, and 30 µg of protein was added to each well of 4–20% Mini-Protean TGX precast acrylamide gels (Bio-Rad). Membranes were probed with HA-tag (Cell Signaling Cat. #C29F4) and GAPDH (Sigma Cat. #G9545) antibodies at 1:2500 and 1:10000 dilutions respectively. Anti-rabbit conjugated HRP secondary antibody (Cell Signaling Cat. #7074 S) was used at 1:2500 dilution for visualization. Blots were developed with HyGlo Quick Spray Chemiluminescent HRP Antibody Detection Reagent (Denville Scientific) and visualized on x-ray film.

### Next-Generation Sequencing

The SpCas9 sequence from an unmutated template, the initial library, and each selection replicate were PCR amplified in a 50 µl reaction with Hot Start Phusion Polymerase (NEB) from 50 ng of plasmid template using 2.5 µl of each 10 µM primer, ProD For and Omega Rev2. The PCR cycles were as follows: 98 °C for 30 seconds, 15 cycles of 98 °C for 10 seconds, 59 °C for 30 seconds, 72 °C for 2 minutes, and a final cycle of 98 °C for 10 seconds, 59 °C for 30 seconds and 72 °C for 5 minutes. The resulting 4.4 kb PCR product was gel extracted and purified with a Zymoclean column. The DNA concentration was quantified with QuantiFluor dsDNA dye (Promega) and used as the template for subsequent subamplicon PCRs.

The entire gene was broken up into 20 subamplicons using a method similar to that described previously^25^. Primers that were used for these amplifications can be found in Supplementary Table 1. Subamplicon PCRs were performed using KOD Hotstart Master Mix (EMD Millipore) with 1 ng template in 24 µl reactions with the following cycling conditions: 95 °C for 2 minutes, 14 cycles of 95 °C for 20 seconds, 54 °C for 20 seconds, 70 °C for 20 seconds and a final denaturation step of 95 °C for 1 minute. PCRs were purified with ZR-96 DNA Clean and Concentrator plates (Zymo Research) and the concentration was determined using QuantiFluor dye. The subamplicons were combined into an equimolar solution which was diluted to 1.55 × 10^5^ single-stranded DNA molecules per subamplicon for the final PCR which added the sequencing indexes.

Index PCRs were performed using KOD Hotstart Master Mix and the appropriate forward and reverse index primers (Illumina) in a total volume of 40 µl with the following cycles: 95 °C for 2 minutes and 24 cycles of 95 °C for 20 seconds, 55 °C for 20 seconds, and 70 °C for 20 seconds. Index PCR reactions were purified using 72 µl of Agencourt Ampure XP Beads (Beckman Coulter) and quantified with QuantiFluor dye. Equal quantities of each reaction were mixed into a final pool and loaded onto a 1.5% agarose gel where the roughly 400 bp band was gel extracted using a Zymoclean column. Sequencing was performed on a NextSeq. 500 with a Mid Output Kit v2 (Illumina) using 2 × 150 bp paired end reads.

### Determination of Mutant Counts from Sequencing Data

In order to get nucleotide counts from subamplicon sequence data, dms_tools version 1.1.3^26^ was modified to include a nucleotide counting feature in dms_barcodedsubamplicons. The count files generated as output were then combined into a large dataframe with added identifying information about the nature of the mutation using custom r and ruby scripts.

### Mutability Score Determination

The number of possible nonsynonymous mutations and the number of these mutations which were significantly increased or decreased after positive selection were determined for each amino acid position throughout the Cas9 gene. Log_2_ fold change was calculated according to equation 1, where *C*_*m*_ indicates mutation counts.

1 $$\frac{{C}_{m}+1}{{C}_{total}+1}={C}_{freq}\,\& \,(\frac{{C}_{fre{q}_{positive}}}{{C}_{fre{q}_{negative}}})=F$$

Weighted averages for increasing or decreasing frequencies were calculated using equation 2.

2 $$\frac{1}{n}\sum _{i=1}^{n}(\,{p}_{w}\times Lo{g}_{2}F)=I\,or\,D\,$$

where *p*_*w*_ was generated by normalizing P values from each group, with the 80^th^ percentile of each group set as the maximum. Increasing and decreasing mutability scores were calculated with equation 3, where *NS* indicates the number of nonsynonymous mutations.

3 $$\frac{I}{{I}_{max}}\times \frac{N{S}_{I}}{N{S}_{total}}={m}_{I}\,or\,\frac{D}{{D}_{max}}\times \frac{N{S}_{D}}{N{S}_{total}}={m}_{D}$$

Final mutability scores at each residue were then calculated by adding the mutability scores of increasing and decreasing mutations, as described in equation 4.

4 $${m}_{I}+{m}_{D}={m}_{Final}$$

These scores were then mapped onto the crystal structure of SpCas9 (PDB ID: 5f9r)^27^.

### Statistical Tests

To assess reproducibility of replicates, the counts from each selection replicate were converted to frequencies avoiding zeroes by adding one to the counts, log_2_ transformed, and compared to each other using Spearman’s rank correlation test.

The Chi Squared test was used to assess the mutation preferences of each domain. Domains boundaries were defined as indicated in^28^.

GFP disruption assays were analyzed by one-way ANOVA followed by linear contrasts. P-values from linear contrasts were adjusted for multiple tests using the Benjamini-Hochberg (BH) procedure.

The counts from each selection replicate were combined and the Fisher’s exact test was used to compare each group. The Fisher test P-values were adjusted using the BH procedure to reduce false discovery rate.

### Availability

Sequencing files have been submitted to the SRA database under the accession number SRP107783.

## Results

### Establishing a Selection System

**Figure 1:**
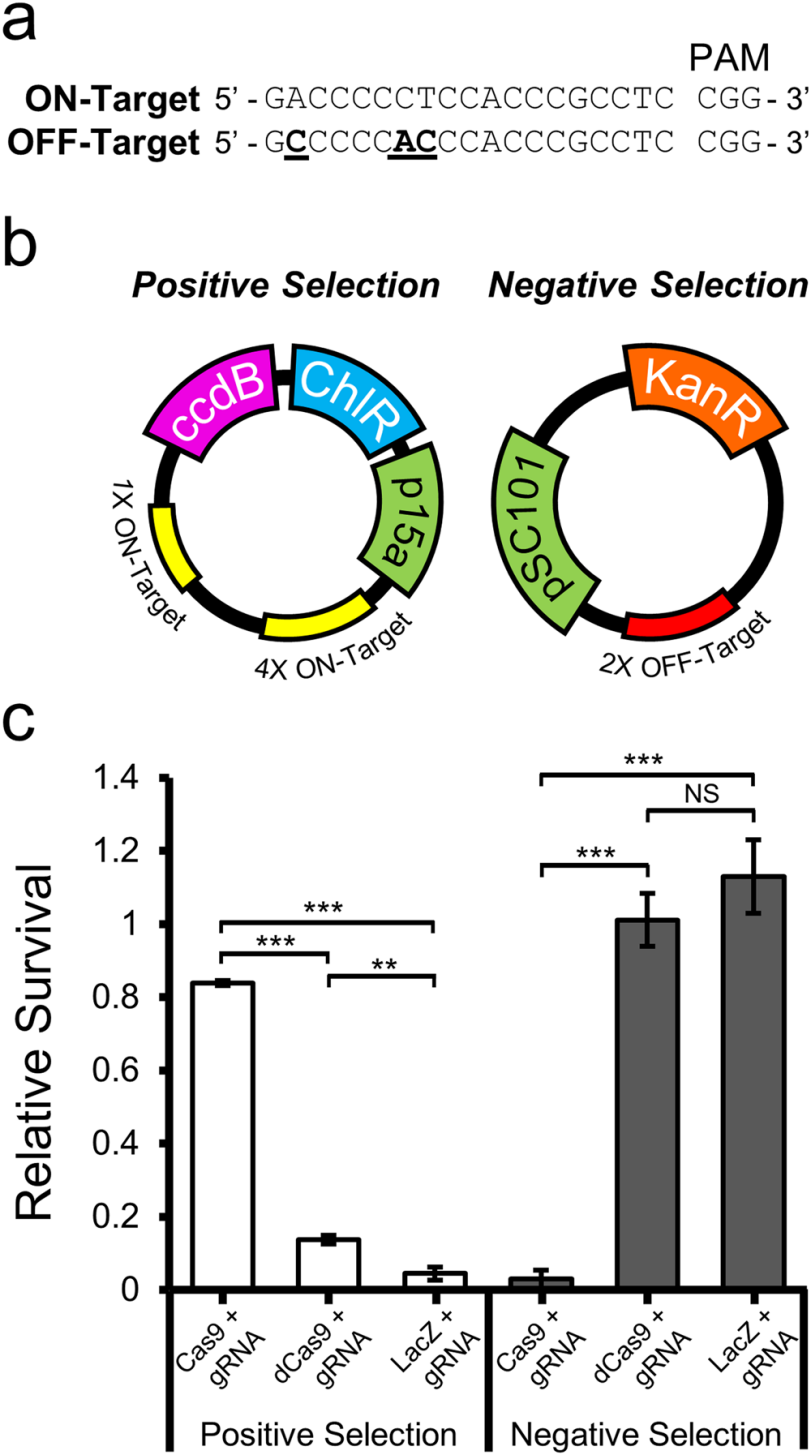
Positive and Negative Selection Systems. (**a**) A target sequence with known off-target activity was chosen to insert into the positive selection plasmid (ON-target). One of the most highly active off-target sequences was inserted into the negative selection plasmid (OFF-target). Mismatched bases are underlined and highlighted in bold. (**b**) Cartoon representation of positive and negative selction plasmids. The positive selection plasmid expresses ccdB under control of the P_tac_ promoter, carries a chloramphenicol resistance gene, and controls copy number with the p15a origin of replication. Yellow boxes indicate sites where four or a single copy of the ON-target sequence have been inserted into the plasmid. The low-copy, negative selection plasmid includes a kanamycin resistance gene and controls its copy number with the pSC101 origin of replication. The red box indicates the insertion of two copies of the OFF-target sequence. (**c**) Selection plasmids were cotransformed with a plasmid expressing the on-target gRNA and either active Cas9, nuclease dead Cas9 (dCas9) or LacZ. Comparison of transformations plated on selective media to control plates were used to assess relative survival. The Cas9 plasmid showed significantly greater rescue on selection media than dCas9 or LacZ (n = 3, error bars indicate S.E.M., one-way ANOVA F_2,6_ = 1181, P = 1.63 × 10^−8^, followed by post-hoc Tukey HSD analysis). Cas9 expressing plasmid showed an inverse response on selection media (n = 3, error bars indicate S.E.M., one-way ANOVA F_2,6_ = 68.63, P = 7.35 × 10^−5^, followed by post-hoc Tukey HSD analysis, P = * < 0.05, ** < 0.01, *** < 0.001, NS = not significant).

We established a selection system in *E. coli* which could differentiate between active and inactive mutant variants of SpCas9. The selection strategy utilized positive and negative selection to provide information about both the on and off-target enzymatic activity of SpCas9 variants. The plasmid used for positive selection incorporated a site whose gRNA had been shown to have a high propensity to cleave similar sequences while the negative selection plasmid harbored an off-target sequence with strong activity in mammalian cells (Fig. 1a,b)^5^. To assess nuclease function, SpCas9 and the gRNA targeting the chosen site were constitutively expressed from a high copy plasmid, compatible for co-transformation with each selection plasmid.

The positive selection plasmid utilizes the tac promoter to inducibly express the DNA gyrase inhibitor, ccdB, in the presence of IPTG. As a result, the inability to remove the plasmid by nuclease cleavage results in cell death. As expected, expression of active SpCas9 and the appropriate gRNA rescues growth of cells containing the positive selection plasmid in the presence of inducer. However, when catalytically inactive SpCas9 (dCas9) was expressed with the gRNA, the majority of cells could not survive on selection plates (Fig. 1c). A small percentage of cells escape cell death with either dCas9 or LacZ expression. This arises from the inability to apply antibiotic selection pressure to maintain the positive selection plasmid while simultaneously selecting for its absence. Although dCas9 appears to provide a slight advantage over LacZ alone, it is unlikely due to transcriptional interference since the target sites are located far from the suicide gene (Supplementary Fig. 1). The higher metabolic burden imparted by the overexpression of the large SpCas9 open reading frame, compared to the much smaller LacZ protein, would put more pressure on the cells to lose the positive selection plasmid and provides a possible explanation for the increased escape rate. However, the large differential response to the presence of an active nuclease complex generates a selection pressure which can identify the disruption of nuclease function.

The opposing arm of selection, negative selection, proceeds through the elimination of the kanamycin resistance conferred by the negative selection plasmid (Fig. 1a,b). Cleavage of the chosen off-target site by unmodified (WT) SpCas9 occurs readily in *E. coli*, as demonstrated by the minimal growth of cells co-transformed with the negative selection and expression plasmids on plates containing kanamycin. Conversely, expression of dCas9 with the gRNA rescues growth on selection plates in the presence of both plasmids (Fig. 1c). These data demonstrate that the off-target sequence can be cleaved efficiently by WT SpCas9 in *E. coli*.

### Production of a SpCas9 Mutant Library

**Figure 2:**
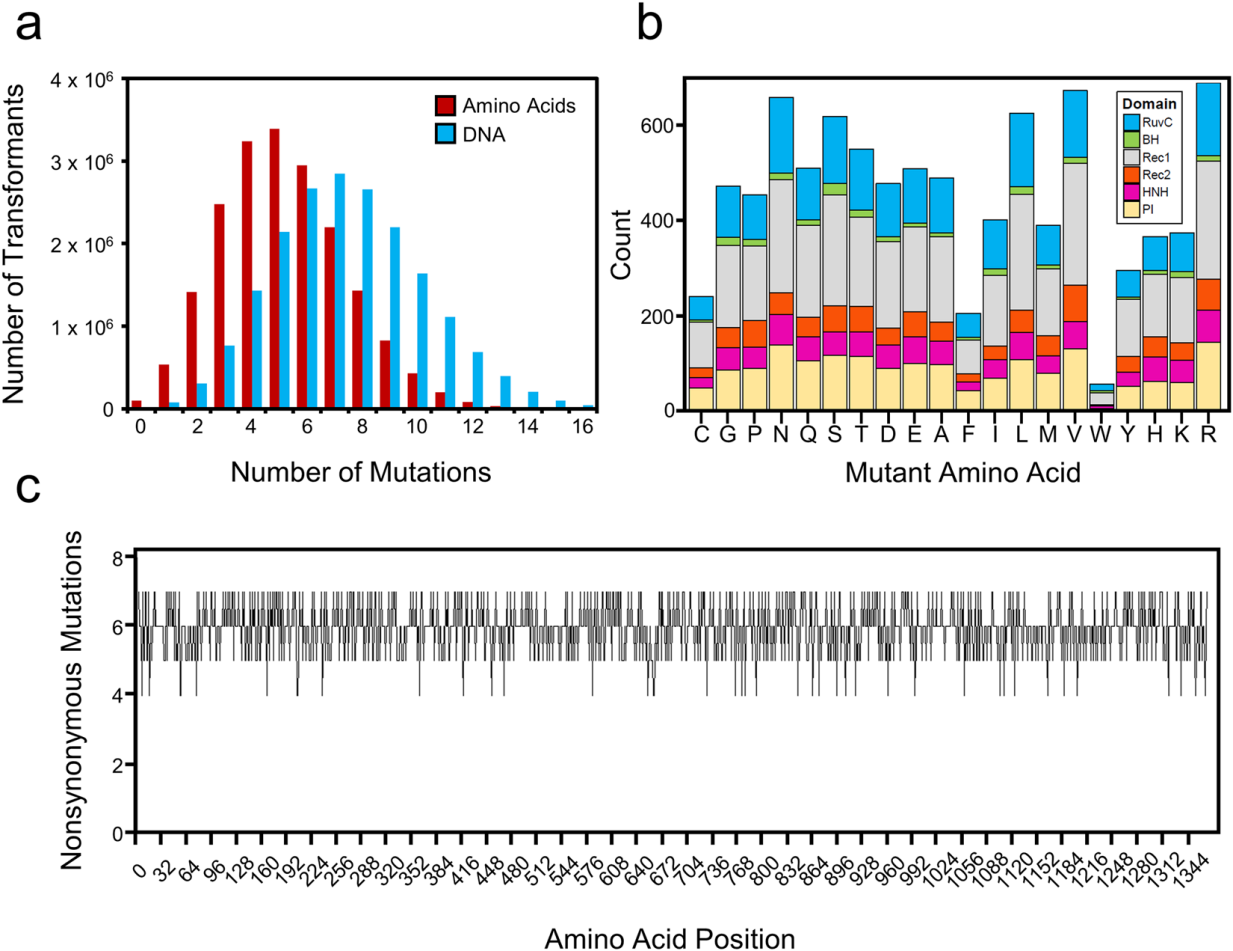
Predicted SpCas9 library composition. (**a**) Red bars represent predicted number of transformants with the indicated number of amino acid mutations. Blue bars represent the number of transformants with the indicated number of DNA mutations. (**b**) Counts of the number of accessed amino acid mutations. Colors indicate the number of each mutation found in the indicated domain. (**c**) Plot of the number of nonsynonymous mutations accessed at each position in SpCas9.

Having generated a selection scheme which could assess the nuclease activity of SpCas9 mutants, we next created a library of variants to screen for functional changes. We randomly mutated the entire SpCas9 gene with error-prone PCR (ep-PCR), resulting in a mutation rate of 0.18%. The mutated genes were cloned into our expression plasmid (Supplementary Fig. 3), generating a library of 1.9 × 10^7^ transformants. Our predictions of the resultant library composition reveal a wide range of total amino acid mutation combinations, with five mutations per gene as the most represented class (Fig. 2a). While not all possible amino acid changes are accessible with ep-PCR at low mutation rates^29^, our particular library contains 8549 possible non-synonymous mutations within the coding region of SpCas9, assuming that nucleotides within the same codon are unlikely to mutate simultaneously (Fig. 2b,c). Indeed, not all amino acid mutations are equally represented, yet each domain contains these mutations at similar frequencies (Fig. 2b and Supplementary Fig. 9).

### Deep Sequencing of Variants Following Selection

Upon completion of the library, we performed negative selection on the population of mutants. We applied negative selection first to enrich for mutants which were inherently more specific than the naturally occurring SpCas9. Following negative selection, the resulting populations of variants were selected for functional nuclease activity using our positive selection system. We utilized deep sequencing at each stage of selection to observe the dynamics of the mutation frequencies for each nucleotide of the SpCas9 coding sequence. Changes in mutation frequency were measured after only one round of each selection to capture a broader range of phenotypic effects. In order to increase the signal above the background mutation rate inherent in NGS, we employed a method refined by Doud *et al*. which partitions the gene of interest into smaller, barcoded fragments that can be re-sequenced, improving the certainty of each nucleotide identity^25^. This approach generated an incredibly low error rate of 0.0043%, as determined by sequencing an unmutated template, likely resulting primarily from the polymerase amplification steps. Demonstrating the robustness of the approach, replicates from each selection strongly correlate (Supplementary Fig. 10).

**Figure 3:**
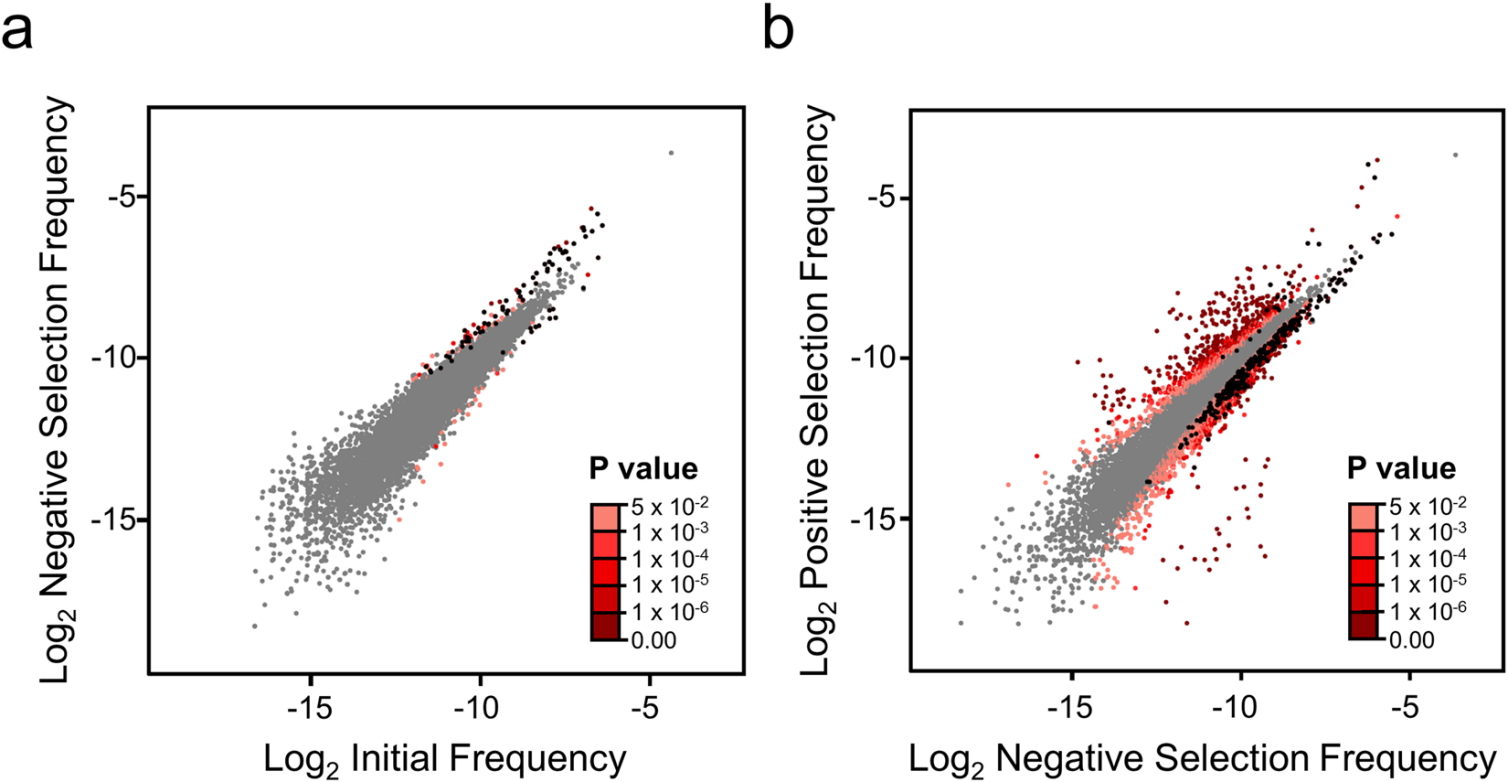
Changes in mutation frequency following selection. (**a**) Mutation frequencies from the initial population plotted against mutation frequencies from negative selection presented as log_2_ transformed frequencies. Shades of red indicate adjusted P values as determined by Fisher’s exact test adjusted using the Benjamini-Hochberg procedure. Stop codons with significantly altered frequencies are colored in black. (**b**) Mutation frequencies from negative selection plotted against positive selection mutation frequencies, log_2_ transformed. P values are calculated as in (**a**).

We evaluated the differences in nucleotide mutation frequency after each selection to infer the functional consequence of the resultant amino acid changes on the nuclease function of SpCas9. Negative selection had a modest effect on the mutation frequencies in the population. In fact, most mutations were not altered after negative selection (Fig. 3a and Supplementary Data 1). Of the mutations with significantly modified frequencies, the majority of nonsynonymous mutations were nonsense mutations (73 of 119). Most nonsense mutations (61 of 73) were enriched as expected. The exceptions, amber stop codons, were likely the result of an amber suppressor mutation in our selection strain (Supplementary Table 4). The remaining nonsynonymous mutations with increased frequency following selection were distributed unequally across the protein. In fact, the majority of these mutations were in the PAM-Interacting (PI) domain (18 of 29). An analysis of the mutations enriched in negative selection showed a slight preference for lysine mutations (Supplementary Fig. 11).

Analysis of the positive selection populations revealed a greater number of significantly altered mutation frequencies (Fig. 3b). As expected, most stop codons were depleted following selection, with opal stops showing the weakest depletion, consistent with its reported weak termination strength (Supplementary Table 4)^30^. The majority of significantly altered mutations were nonsynonymous amino acid substitutions (3248 of 3453). Also of note, the majority of significantly altered nonsynonymous mutations decreased in frequency following selection (2112 of 3248) implying that the bulk of mutations disrupt protein function.

Using the proportion of significantly enriched or depleted mutations at each site and the effect size of each mutation, we generated a mutability score for each amino acid in SpCas9 as a measure of the importance of the identity of each residue on nuclease activity. The spatial organization of residues with high or low scores should indicate regions which, when altered, presumably disrupt or enhance nuclease function (Supplementary Fig. 12).

**Figure 4:**
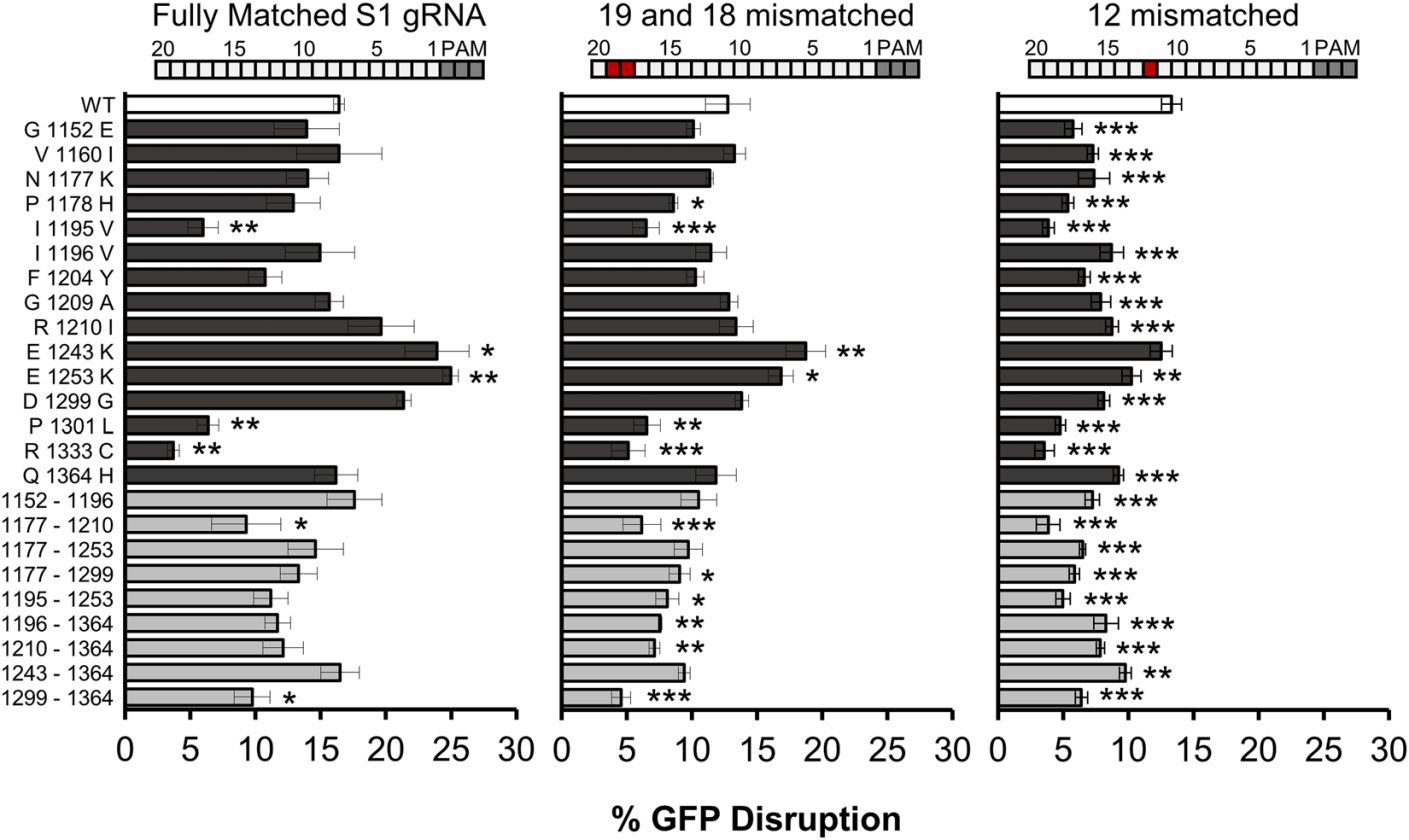
Activity of negative and positive selection enriched mutants. Mutations which were enriched following both positive and negative selection were expressed in mammalian cells, targeting a chromosomally integrated copy of GFP. Differences in on-target activity were assessed with the site 1 (S1) gRNA (n = 3, error bars indicate S.E.M., one-way ANOVA F_24,50_ = 6.34, P = 2.1 × 10^−8^, followed by post hoc linear contrasts). We tested the ability of mutants to cleave a doubly mismatched S1 target (n = 3, error bars indicate S.E.M., one-way ANOVA F_24,50_ = 9.16, P = 3.4 × 10^−11^, followed by post hoc linear contrasts) and a singly mismatched target (n = 3, error bars indicate S.E.M., one-way ANOVA F_24,50_ = 11.37, P = 5.7 × 10^−13^, followed by post hoc linear contrasts, P = * < 0.05, ** < 0.01, *** < 0.001). Bars above each graph indicate the positions of mismatches with red colored boxes.

Consistent with previous characterization, at least one mutation of D10 and several H840 mutations were significantly decreased following selection (D10G, H840Q, H840R, and H840L) and had low mutability scores (Supplementary Figs 13 and 14)^31^. Furthermore, two mutations of the PAM guanine binding amino acid, R1335, were significantly depleted following selection, resulting in a low mutability score as well (Supplementary Fig. 15). The other PAM contacting amino acid, R1333, was significantly depleted when mutated to histidine but was enriched for cysteine substitution. We determined that R1333C was functional on the selected target in bacterial cells, but could no longer function in mammalian cells when targeted with a different gRNA, suggesting a sequence specific tolerance of this substitution (Fig. 4 and Supplementary Fig. 16). Interestingly, it has been observed that R1333 mutations can retain function on some targets with as yet unknown preference^32^. Alpha helical linkers connecting the RuvC and HNH domains have been shown to be important for allosteric regulation of non-target strand cleavage^33^. Congruent with the importance of these helices in mediating dsDNA cleavage, the majority of mutations in this region showed significant depletion in our positive selection data and consequent low mutability scores (Supplementary Figure 13 and Supplemental Data 1). These data demonstrate that disruption of important residues and features perform as predicted in our selection system.

We evaluated the frequencies of mutation types which were enriched and depleted after positive selection in an effort to identify any trends in amino acid enhancement or disruptive properties (Supplementary Fig. 17). Glutamic acid appeared to enhance Cas9 function most frequently, while tryptophan most commonly disrupted protein function when normalized to the number of instances of each mutation (Supplemental Fig. 17a). Glutamine was the most represented amino acid among the mutations in the top ten percent of enriched mutations. Interestingly, the majority of these were substitutions for positively charged residues (7 of 10) (Supplementary Fig. 17d). When looking at the significantly depleted population, arginine substitutions were the most represented mutations in the bottom ten percent (Supplementary Fig. 17d).

### Mutation Tolerance of Protein Domains

**Figure 5:**
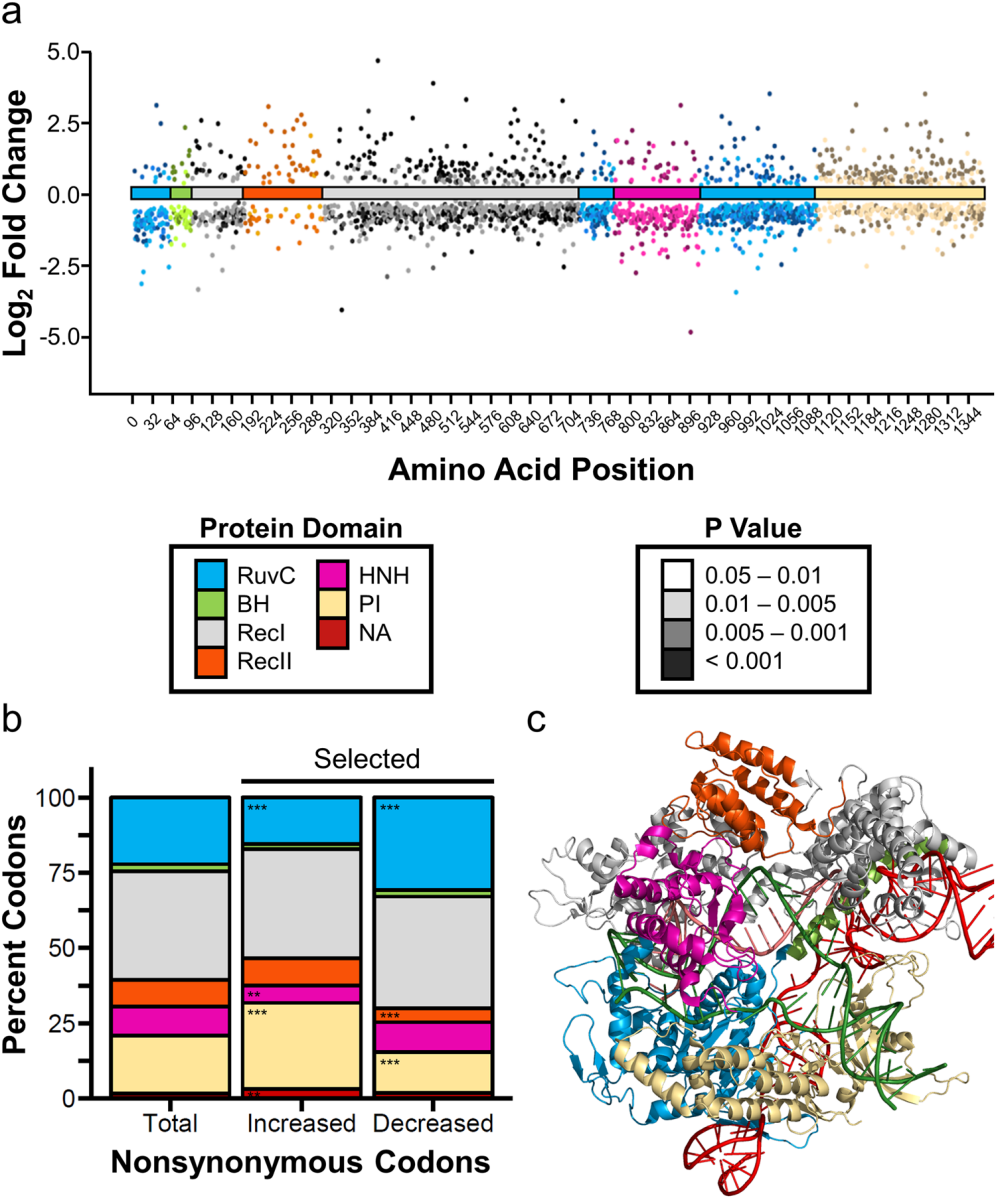
Domains have varied responses to mutation. (**a**) The log_2_ transformed fold change in mutation frequency following positive selection is plotted across the length of SpCas9. Each point represents a unique nonsynonymous mutation that was significantly enriched or depleted following selection. Points are colored by protein domain. The shade of the color indicates P value as calculated by Fisher’s exact test adjusted using the Benjamini-Hochberg procedure. (**b**) Nonsynonymous codons that resulted from one nucleotide change were counted for each structural domain and represented as a percent of all possible nonsynonymous codons within the open reading frame of the protein (Total). Similarly, nonsynonymous codons which were significantly enriched (Increased) or depleted (Decreased) following selection were counted and represented as a percent of all significantly altered codons of each respective class. Chi-squared analysis was performed on the frequencies of significantly altered codons to determine which domains were more or less tolerant of mutations (P = ** < 0.01, *** < 0.001). (**c**) Cartoon structure of SpCas9 bound to gRNA and dsDNA based on PDB: 5f9r (ref.^27^). Domains are colored as in (**a**) and (**b**) for reference.

In order to understand which functional domains of SpCas9 best tolerate mutation, we asked whether the proportion of significantly enriched or depleted mutations in each domain following positive selection differed from the proportion of possible nonsynonymous codons for that domain. We excluded from the analysis known translational effects resulting from nonsense, start codon, and synonymous mutations in order to focus on changes which had the potential to directly modify enzymatic or structural properties. Interestingly, the population of mutants which were enriched following positive selection had a significantly lower proportion of mutants in the RuvC and HNH domains. Conversely, this population had a higher proportion of mutants in the PI domain and the artificial domain containing the NLS and HA tag. When we evaluated the population of mutants which were depleted after positive selection, we found that the proportion of mutants in the RuvC domain were significantly increased while the proportion of mutants in the REC2 and PI domain were decreased (Fig. 5a,b). These data are consistent with the role of the RuvC and HNH domain in mediating DNA strand cleavage. Further, the domains that were most tolerant of mutation, the REC2 and PI domains, are also the least conserved domains in Cas9 families. In fact, the REC2 domain has been found to be dispensable for SpCas9 cleavage, although with a reduction in activity^28^. Indeed, replacement of the Rec2 domain with BCL-XL produces a functional Cas9^34^.

### Identification of Functionally Distinct Mutants

**Figure 6:**
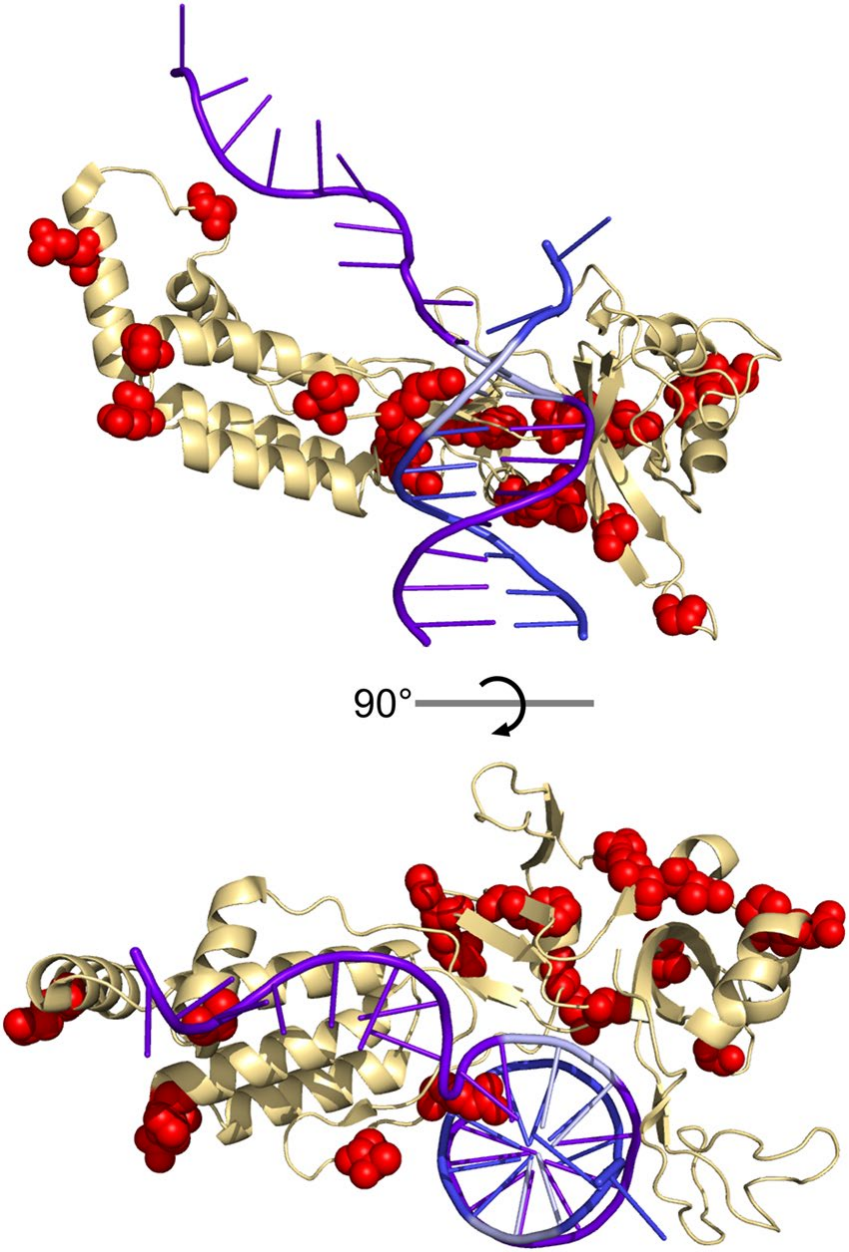
Specificity enhancing mutations map to the PI domain. Cartoon structure of the PI domain of SpCas9 bound to dsDNA based on PDB: 5f9r (ref.^27^). Residues that were found to have mutations which were enriched in both positive and negative selection are shown as red spheres. The PAM nucleotides are colored in pale blue.

We investigated the mutations whose frequencies were increased following both negative and positive selection in an attempt to identify mutations which could confer increased target fidelity. The mutations of this class were almost exclusively in the PI domain (18 of 19) with the only other mutation (K44R) in the RuvC domain. When we mapped these mutated residues onto SpCas9’s crystal structure, we noticed that they formed two distinct clusters, either encircling the PAM-containing dsDNA or in the region interacting with the unwound non-target strand (Fig. 6). We generated single PI domain mutations from each of these sets to investigate their effect on nuclease activity in mammalian cells. We employed a GFP disruption assay similar to one previously reported as a measure of nuclease activity^35^.

Most of the mutants that were predicted to retain activity were able to cleave chromosomally integrated GFP in mammalian cells with efficiency similar to WT SpCas9 (Fig. 4). The mutations that were close to the PAM dsDNA tended to have activity similar to WT SpCas9, while the mutations clustered near the non-target DNA tended to have increased activity (Fig. 4). At least one of the mutants that was not efficient in mammalian cells (R1333C) showed robust cleavage of the selection plasmid in bacterial cells suggesting that there may be a guide or species specific preference for this and possibly other mutations (Supplementary Fig. 16). One of the other notable discrepancies, P1301L, had poor expression, which likely accounts for its low activity (Supplementary Fig. 18). We assessed the ability of the single mutants to cleave with single or double mismatched gRNAs. The gRNA with a single mismatch more proximal to the PAM was cleaved at a significantly lower rate by almost all of the mutants (one-way ANOVA F_24,50_ = 11.37, P = 5.7 × 10^−13^), indicating an increase of specificity at this location (Fig. 4). However, a 5′ double-mismatched gRNA was cleaved at the same rate as WT (Fig. 4). These improvements on specificity are unlikely to be caused by reduced protein expression as many variants with improved off/on target ratios have similar expression levels as WT SpCas9 (Supplementary Fig. 20 and Supplementary Fig. 18).

The improvement on specificity imparted by the single mutations prompted us to consider if further improvements could be made by combining these mutations. A few of the doubly mutated proteins were still able to function and showed a significant reduction in activity when targeted with the double-mismatched gRNA (Fig. 4, one-way ANOVA F_24,50_ = 9.16, P = 3.4 × 10^−11^). We investigated whether the improvements to specificity were target dependent by measuring the overall activity and mismatch tolerance of the best performing mutants on a set of different target sequences. With these targets, many of the mutants had generally lower activity than WT SpCas9 (Supplementary Fig. 19b). However, the Q1364H mutation and the doubly mutated G1152E- I1196V variants retained on-target activity and demonstrated mismatch-specific target selectivity improvements with single mismatched gRNAs (Fig. 4 and Supplementary Fig. 19a).

**Figure 7:**
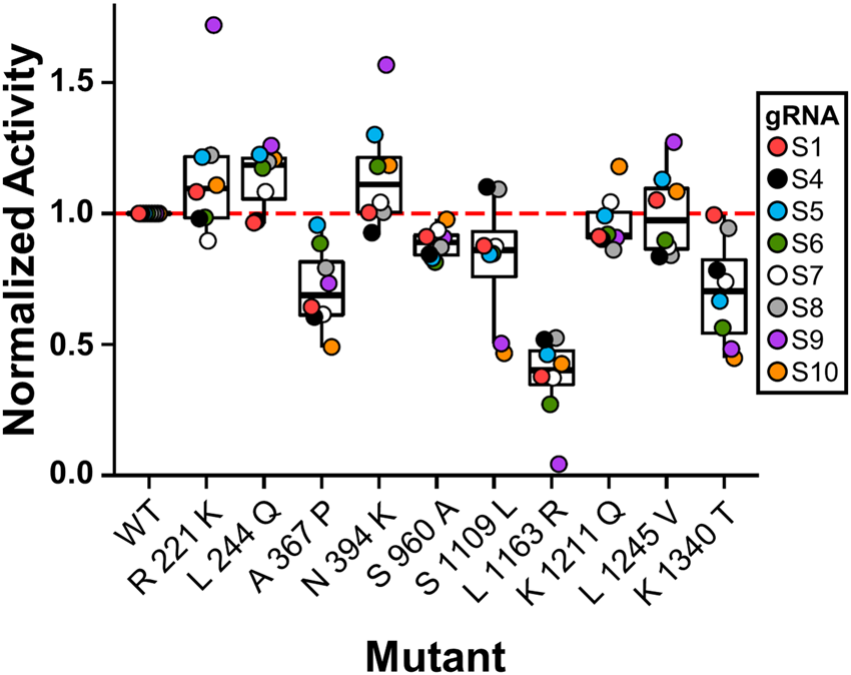
Activity of additional positive selection enriched mutants. Ten mutations which were enriched after positive selection were tested for their ability to cleave chromosomally integrated GFP using eight different gRNAs. Their activity was normalized between WT SpCas9 activity with each gRNA and a non-targeting gRNA.

To demonstrate that our data set could be used to find mutants with improved nuclease activity, we chose an additional ten mutants to test in mammalian cells which increased in frequency following positive selection, but not necessarily negative selection. We included several mutations of residues with high calculated mutability scores to evaluate the consistency of this data (Supplementary Figs 21 and 15). Many of these are located in the Rec2 and PI domains since they appeared most tolerant of mutation according to our analysis (Fig. 4). We tested their ability to cleave with eight different gRNAs targeting GFP in our disruption assay. Most of these single mutants (9 of 10) retained at least seventy percent WT SpCas9 activity with half of the gRNAs tested (Fig. 7). The mutants with the lowest activity also had reduced expression compared to WT SpCas9 indicating that the reduction in activity may be a consequence of poor translation or protein stability rather than nuclease activity (Supplementary Fig. 22). Combined with the fifteen mutants previously tested that were enriched in both positive and negative selection (Fig. 4), over eighty percent of single mutations (21 of 25) which increased in frequency following positive selection demonstrate the same trend in activity in mammalian cells, indicating that changes in frequency in the bacterial selection experiments are good proxies for activity in mammalian cells, and translational differences or stability changes, rather than loss of enzymatic activity, may play a role in some cases where activity appears reduced (Supplementary Data 1 and Supplementary Fig. 22).

**Figure 8:**
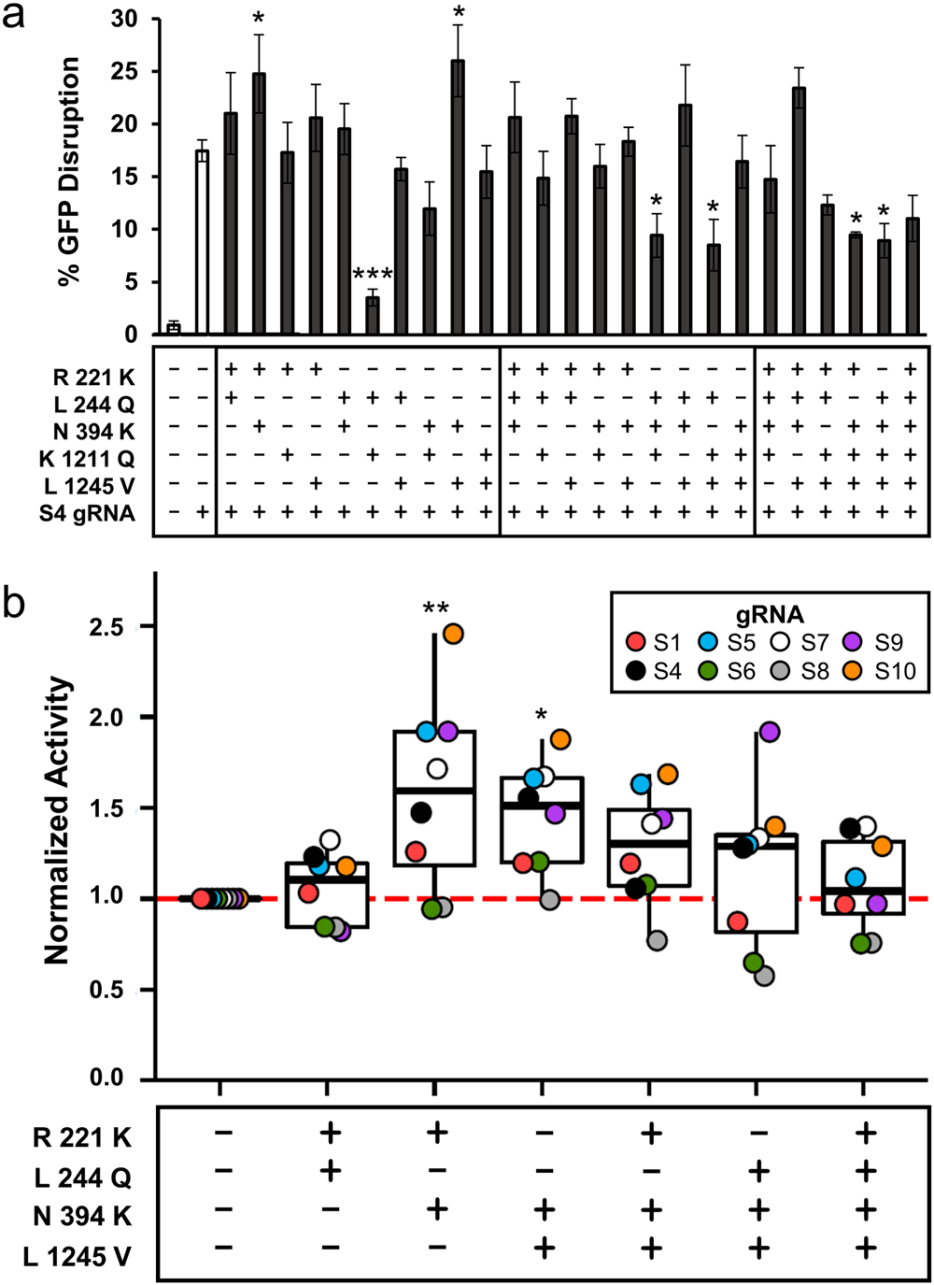
Activity of positive selection enriched mutant combinations. (**a**) Combinations of mutations that had retained activity in mammalian cells as single mutants were compared to WT SpCas9’s ability to cleave with a gRNA targeting GFP site 4 (S4) (n = 3, error bars indicate S.E.M., one-way ANOVA F_25,52_ = 5.07, P = 4.0 × 10^−11^, followed by post hoc linear contrasts). (**b**) Top performing combinations were targeted against chromosomally integrated GFP with a total of eight gRNAs and their activity was normalized between WT SpCas9 with each gRNA and a non-targeting gRNA (one-way ANOVA F_6,161_ = 3.65, P = 0.0020, followed by post hoc linear contrasts, P = * < 0.05, ** < 0.01, *** < 0.001).

As some of the mutations appeared to generally increase the nuclease activity of SpCas9, we made combinations of the five single mutants with the highest retained activity across multiple gRNAs. As an initial assessment, we targeted these combinatorial mutants to cleave GFP with two different gRNAs. From these experiments, combinations which contained the K1211Q mutation tended to have lower activity than the other mutation combinations (Fig. 8a and Supplementary Fig. 23). Some of the combinations had a significant increase in activity compared to WT SpCas9 (Fig. 8a, one-way ANOVA F_25,52_ = 5.07, P = 4.0 × 10^−11^). We further tested the best performers with the remaining set of gRNAs targeting GFP to understand if the increases in activity were a general property of these mutants. The combination of R221K and N394K showed the largest significant increase in activity followed by the N394K and L1245V combination, with a median activity of 1.5 times that of WT SpCas9 (one-way ANOVA F_6,161_ = 3.65, P = 0.0020). Mixing all three of these mutations reduced the activity to near WT SpCas9 levels, while the remaining combinations showed no significant difference to WT SpCas9 (Fig. 8b). Nonetheless, combinations of these mutations were generally well tolerated by the protein and in some cases enhance the nuclease activity.

## Discussion

The toolbox of CRISPR-associated RGENs continues to grow at a rapid pace^15,18–20^. Before clinical application can occur with these new systems, each protein will require careful characterization and refinement. Refining the activity of RGENs to meet the complex challenges of therapeutic applications requires in-depth knowledge of their structure and consequent functions imparted by those intrinsic features. The work we present here demonstrates the facility with which one can define important amino acid residues within an RGEN through DMS experiments using SpCas9 as a model. Our DMS data demonstrate changes in mutation frequency which correlate well with known features of SpCas9. Examination of the data reveals trends of differential mutation tolerance among the distinct functional domains. We investigated some of the unique mutations enriched during the screening process for their activity in mammalian cells and determined that they have similar effects on the protein function, in some cases irrespective of gRNA while other effects appear to be gRNA dependent. The insights from these experiments highlight regions of the protein which contribute to the enzymatic activity of SpCas9. Mutation tolerance and enzymatic alterations from a subset of mutations suggest new strategies which will help guide further SpCas9 engineering.

Further, we show how including additional selection pressure can help resolve a more complex phenotype like specificity. Using two distinct types of selection, we show that, unexpectedly, mutations to the PI domain improve mismatch discrimination for bases outside of the classical seed sequence, albeit in a target-dependent fashion, as demonstrated by the decreased cleavage of a single mismatched target at position 12 for the S1 target and positions 15 and 19 for the S7 target (Fig. 4 and Supplementary Figs 19a and 20)^5,31^. The sequence-specific improvements may be a result of the mismatched target we chose to use in our selection (Fig. 1a). Perhaps including multiple mismatched substrates would further improve the ability to detect additional specificity enhancing mutants. The inclusion of an array of single mismatches on the negative selection plasmid could increase selection pressure and reveal novel specificity enhancing mutations that were not present in our screen. However, the increased fidelity resulting from the PI mutations found by our screen identifies the PI domain as a potential target for further improvements.

Changes to the PI domain surrounding the PAM DNA could affect the stability of DNA unwinding, reducing excess binding energy thereby collapsing the R-loop more easily when bases fail to pair. Mutations near the non-target strand may similarly destabilize protein-DNA interactions which would favor unwinding only in the presence of more perfectly matched target. While further experiments will need to be done to verify this hypothesis, these ideas are supported by mutations to the phosphate-lock altering specificity *in vitro* which was part of the hypothesis driving the generation of high-fidelity SpCas9 variants, although recent work published while this manuscript was in revision uncovered an alternative mechanism for these variants^11,12,36,37^. Our selection was not able to identify the specificity enhancing mutations that were reported in these previous engineering efforts^11,12^. Since we relied on ep-PCR to generate our library, alanine mutations were not generated at these sites. The mutations that were generated at these positions appear to compensate for the WT amino acid. Future DMS experiments could overcome this using saturating mutagenesis to build more complete variant libraries^38^. The mutations we have identified have modest effects on specificity compared to the dramatic reduction in off-target cleavage imparted by SpCas9-HF and eSpCas9^11,12^. However, since these mutations cluster in a divergent domain, they likely operate through a unique mechanism. The combination of these PI mutations with known specificity enhancing mutations may be able to further refine SpCas9 off-target activity and will be an interesting area for future investigation.

Additional mutations in the REC and PI domains were shown to increase nuclease activity across several different gRNAs. Surprisingly, only one of these mutations appears positioned close to bound DNA in a dsDNA bound crystal structure (Supplementary Fig. 24)^27^. Although the possibility cannot be excluded that these residues directly contact DNA during the transition from the unbound state to activated R-loop formation, our data suggests that mutations of these residues may influence distant DNA interactions, altering the dynamics of nuclease activation, and facilitating cleavage. The Rec2 domain has been shown to regulate access of the HNH domain to its DNA substrate^37^. The R221K and N394K mutations, positioned at the interface of the Rec1 and Rec2 domains, may alter this dynamic, facilitating HNH positioning and consequent DNA cleavage. The L1245V mutation, by shortening the R-group and removing steric hindrance, could provide greater flexibility to accommodate the unbound DNA strand during R-loop formation. Further experiments should seek to confirm these hypotheses as they may provide a general mechanism that could yield more active variants.

It is likely that the observed discrepancies in human cell nuclease activity stem from prokaryotic and eukaryotic differences in DNA accessibility. Consistently, it has been observed that many Cas9 orthologs which cleave DNA efficiently *in vitro* perform poorly in mammalian systems^15,39^. The apparent differential enzymatic activity *in vivo* appears to result from chromatin impediment and the differing efficiency with which Cas9 orthologs can displace nucleosomes^39–41^. Consequently, changes in nuclease activity may not translate directly from a bacterial cell into a mammalian cell which has complex, chromatin-architectural constraints. Performing a selection in mammalian cells rather than *E. coli* could potentially overcome this limitation, but mammalian screens have much lower capacities and would not be able to assay as many mutations simultaneously. Despite these known differences between prokaryotic and eukaryotic cell DNA architecture, our prokaryotic data identified mutations with similar properties in mammalian cells as demonstrated by our data characterizing mutations with specificity and activity enhancing properties (Figs 4, 7, and 8).

Our data provide a starting point for understanding the role of each amino acid in gRNA-targeted SpCas9 nuclease activity. Further selections using a nuclease screen in combination with a DNA-binding screen like the one previously described^42^ or a gRNA-binding assay may allow one to parse out specific amino acids involved in each step of SpCas9 activation. Integration of information from each screen will allow researchers to create a detailed map of important regions which contribute to specific functions in the multi-step DNA cleavage process of RGENs. Additional screens of targeted, saturating libraries of the REC and PI domains may yield variants with even more improved functions. Furthermore, performing selections with domain specific libraries would allow epistatic interactions to be investigated, revealing promising mutation combinations which may not be viable as single mutations. The application of similar strategies to newly discovered RGENs will facilitate rapid maturation of each nuclease system into a mature technology.

## Electronic supplementary material

Supplementary Figures

Supplementart Data 1
