# Supplementary material for: Deep mutational scanning of S. pyogenes Cas9 reveals important functional domains
Source: Sci Rep. 2017 Dec 4;7:16836. doi: 10.1038/s41598-017-17081-y (PMC5715146; doi:10.1038/s41598-017-17081-y)
Supplement: Supplementary file 1 — Supplementary Figures [file 41598_2017_17081_MOESM1_ESM.pdf]

Deep mutational scanning of *S. pyogenes* Cas9 reveals important functional domains

Jeffrey M Spencer[1,2] and Xiaoliu Zhang[1,2,*]

[1] Department of Biology and Biochemistry, University of Houston, Houston, Texas, 77204, USA

[2] Center for Nuclear Receptors and Cell Signaling, University of Houston, Houston, Texas, 77204, USA

* To whom correspondence should be addressed. Email: xzhang5@central.uh.edu

Supplementary Figure 1

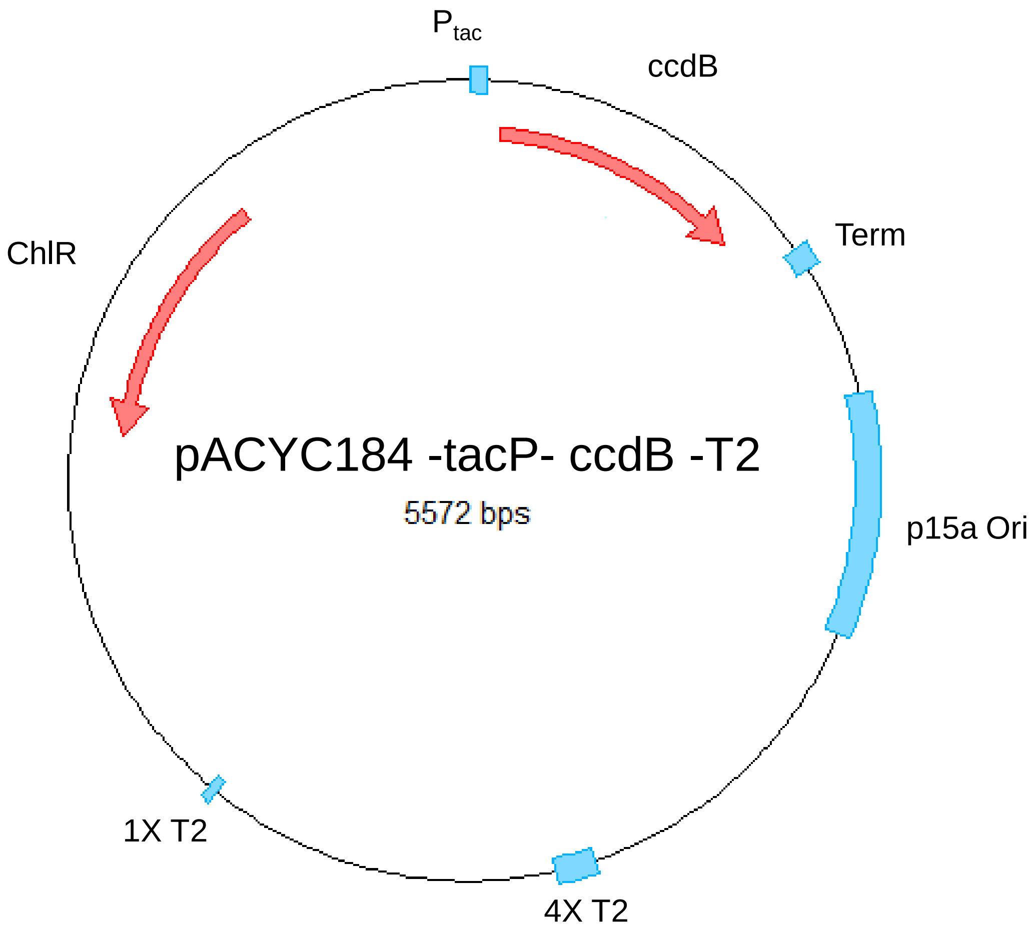

Supplementary Figure 1: pACYC184-tacP-ccdB-T2 Plasmid Map. This plasmid was used for positive selection. Multiple gRNA-targeted sites (T2) were inserted at different locations in the plasmid to facilitate degradation of the plasmid following Cas9 cleavage. The P_{tac} drives IPTG dependent ccdB expression. The p15a promoter maintains the plasmid at moderate copy levels.

Supplementary Figure 2

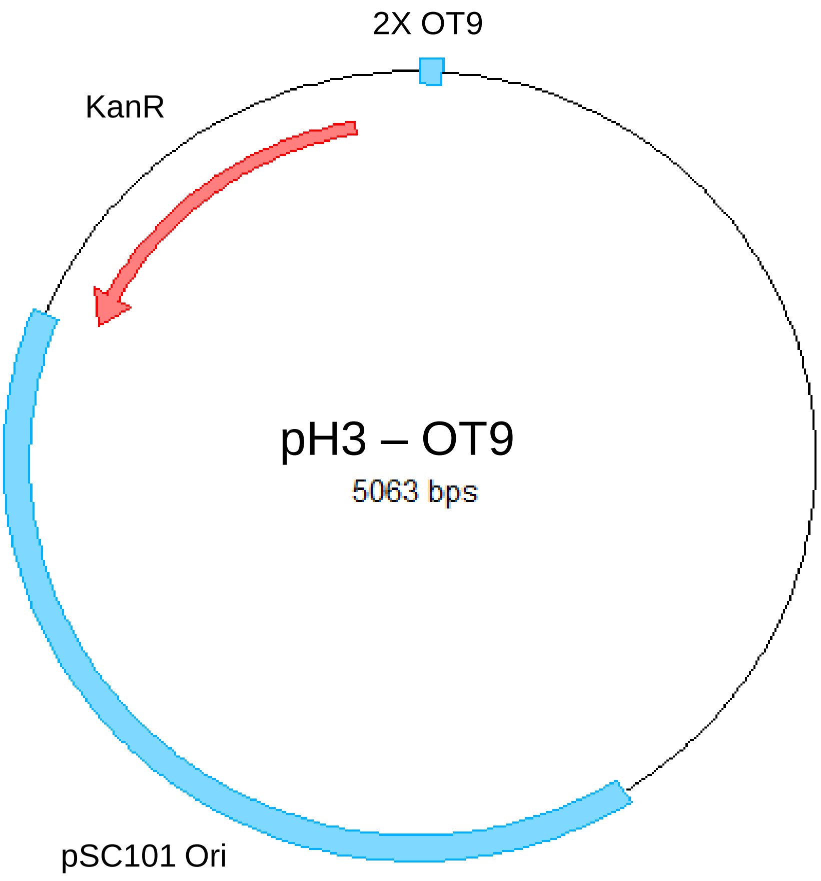

Supplementary Figure 2: pH3-OT9 Plasmid Map. This plasmid was used for negative selection. Two gRNA off-target sites (OT9) were inserted in the plasmid to facilitate Cas9 cleavage. The pSC101 origin of replication maintains the plasmid at a low copy number.

Supplementary Figure 3

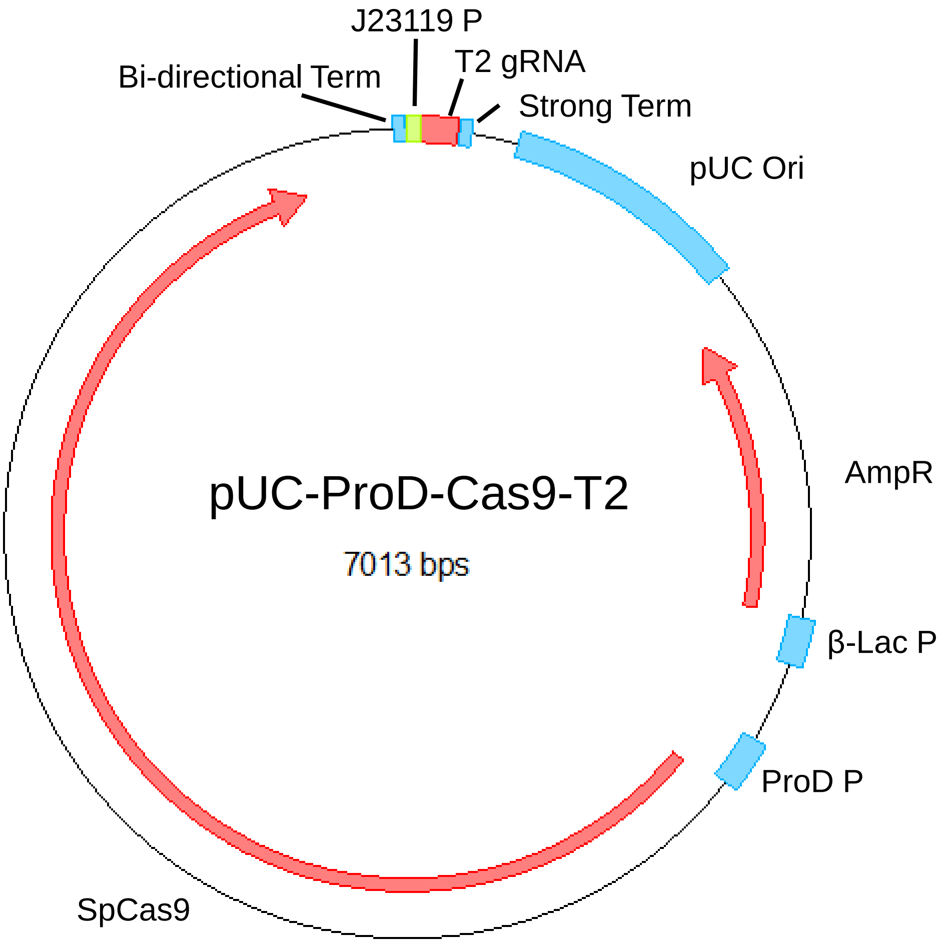

T2 Protospacer: GACCCCCTCCACCCCGCCTC

Supplementary Figure 3: pUC-ProD-Cas9-T2 Plasmid Map. This plasmid was used to express SpCas9 and a targeting gRNA (T2). The synthetic, insulated promoter, ProD, constituvely drives SpCas9 expression. The short, synthetic promoter, J23119, has a defined transcription initiation site making it ideal for gRNA expression.

Supplementary Figure 4

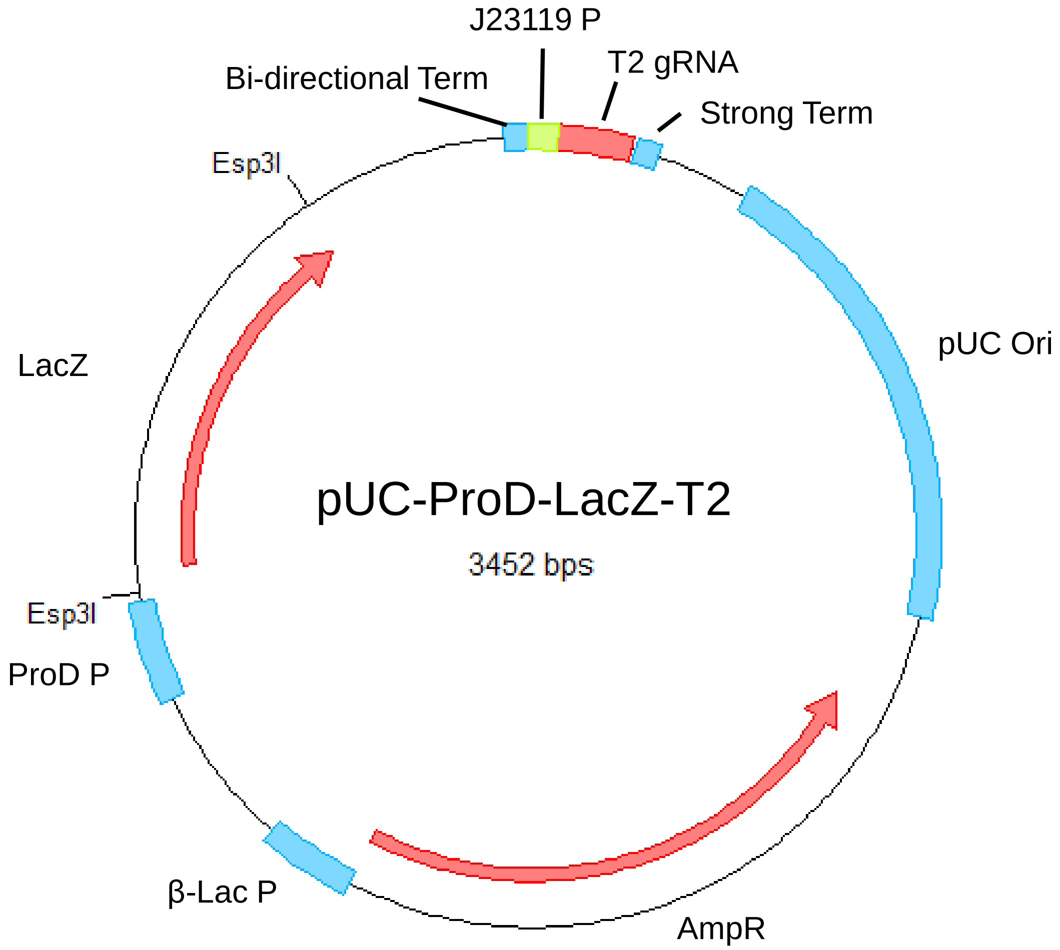

T2 Protospacer: GACCCCCTCCACCCCGCCTC

Supplementary Figure 4: pUC-ProD-LacZ-T2 Plasmid Map. This plasmid was used to clone the SpCas9 mutant library. Esp3I sites allow for efficient Golden Gate Cloning of PCR amplicons into the expression cassette.

Supplementary Figure 5

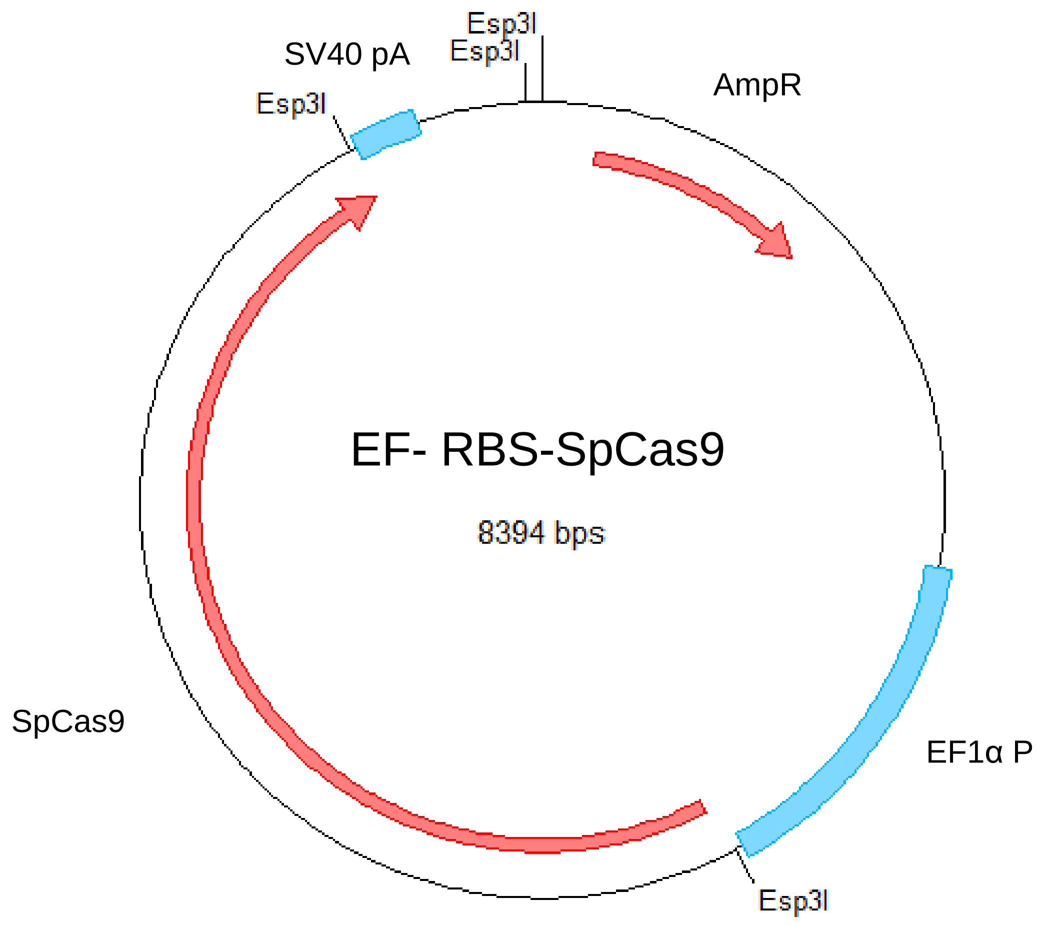

RBS –
5’ – TCACACAGGAAACC – 3’

Supplementary Figure 5: EF-RBS-SpCas9 Plasmid Map. This plasmid was generated to create a SpCas9 template with a prokaryotic RBS that was distinct from the desired final product in order to reduce unmutated template background. Additionally, Esp3I sites required for Golden Gate cloning were added to simplify the post-PCR cloning procedure.

Supplementary Figure 6

Human Codon Optimized SpCas9 Coding Sequence

NLS HA Tag

ATGGATAAAAAATACTCAATCGGGCTGGACATCGGGACCAACTCAGTGGGCTGGGCAGTCATCACAGACGAATACAAAGTCC
CAAGCAAGAAGTTCAAGGTGCTGGGGAACACCGATCGCCACAGTATCAAGAAAAATCTGATTGGGGCCCTGCTGTTCGACTC
AGGAGAGACAGCTGAAGCAACTCGGCTGAAAAGAACAGCTCGGAGAAGGTATACTCGCCGAAAGAATCGGATCTGCTACCTG
CAGGAGATTTTCAGCAACGAAATGGCCAAGGTGGACGATAGCTTCTTTCATAGACTGGAGGAATCCTTCCTGGTCGAGGAAG
ATAAGAAACACGAGAGGCATCCCATCTTTGGCAACATTGTGGACGAGGTCGCTTATCACGAAAAGTACCCTACTATCTATCAT
CTGCGGAAGAAACTGGTGGACTCTACCGATAAAGCAGACCTGAGACTGATCTATCTGGCCCTGGCTCACATGATTAAGTTCAG
GGGACATTTTCTGATCGAGGGCGATCTGAACCCAGACAATAGCGATGTGGACAAGCTGTTCATCCAGCTGGTCCAGACATAC
AATCAGCTGTTTGAGGAAAACCCCATTAATGCATCAGGAGTGGACGCAAAAGCCATCCTGAGCGCCCGCCTGTCTAAGAGTC
GGAGACTGGAGAACCTGATCGCTCAGCTGCCAGGCGAAAAGAAAAACGGACTGTTTGGCAATCTGATTGCACTGTCCCTGGG
GCTGACACCCAACTTCAAGTCTAATTTTGATCTGGCCGAGGACGCTAAACTGCAGCTGTCTAAGGACACTTATGACGATGACC
TGGATAACCTGCTGGCTCAGATCGGCGATCAGTACGCAGACCTGTTCCTGGCCGCTAAGAATCTGTCTGACGCCATCCTGCT
GAGTGATATTCTGAGAGTGAACACCGAGATTACAAAAGCCCCCCTGAGCGCCAGCATGATCAAGCGGTACGACGAGCACCAT
CAGGATCTGACCCTGCTGAAGGCTCTGGTGCGGCAGCAGCTGCCTGAGAAGTACAAAGAAATCTTCTTTGATCAGAGCAAGA
ATGGGTACGCCGGATATATTGACGGCGGGGCTTCCCAGGAGGAATTCTACAAGTTTATCAAACCTATTCTGGAGAAGATGGAC
GGCACCGAGGAACTGCTGGTGAAACTGAATAGGGAAGACCTGCTGAGGAAGCAGCGCACATTTGATAACGGGAGCATCCCT
CACCAGATTCATCTGGGAGAGCTGCACGCCATCCTGAGGCGCCAGGAAGACTTCTACCCATTTCTGAAGGATAACAGGGAGA
AGATCGAAAAAATTCTGACCTTCCGCATTCCCTACTATGTGGGACCTCTGGCAAGAGGCAATAGTAGGTTTGCCTGGATGACA
AGAAAATCAGAGGAAACAATCACTCCCTGGAACTTCGAGGAAGTGGTCGATAAGGGCGCTTCCGCACAGTCTTTCATTGAGA
GGATGACAAACTTCGACAAGAACCTGCCAAACGAAAAAGTGCTGCCCAAGCACTCTCTGCTGTACGAGTATTTCACCGTCTAT
AACGAACTGACAAAGGTGAAATACGTCACTGAGGGCATGCGCAAGCCTGCCTTCCTGAGCGGGGAACAGAAGAAAGCTATCG
TGGACCTGCTGTTTAAAACCAATCGGAAGGTGACAGTCAAGCAGCTGAAAGAGGACTATTTCAAGAAAATTGAATGTTTCGATT
CTGTGGAGATCAGTGGAGTCGAAGACCGGTTCAACGCCAGCCTGGGCACCTACCACGATCTGCTGAAGATCATTAAGGATAA
AGACTTCCTGGACAACGAGGAAAATGAGGATATCCTGGAAGACATTGTGCTGACCCTGACACTGTTTGAGGATAGGGAAATG
ATCGAGGAACGCCTGAAGACTTATGCCCATCTGTTCGATGACAAAGTGATGAAACAGCTGAAGCGACGGAGATACACCGGGT
GGGGACGACTGTCCCGGAAGCTGATCAATGGCATTAGAGACAAACAGAGCGGGAAGACTATCCTGGACTTTCTGAAATCCGA
TGGCTTCGCCAACAGGAACTTCATGCAGCTGATTCACGATGACAGCCTGACCTTCAAAGAGGATATCCAGAAGGCACAGGTG
TCCGGCCAGGGGGACTCTCTGCACGAGCATATCGCAAACCTGGCCGGATCCCCTGCCATCAAGAAAGGCATTCTGCAGACC
GTGAAGGTGGTCGACGAGCTGGTGAAAGTCATGGGGCGCCATAAGCCAGAAAACATCGTGATTGAGATGGCCCGAGAAAAT
CAGACCACACAGAAAGGACAGAAGAACTCAAGGGAGCGCATGAAACGCATCGAGGAAGGGATTAAGGAACTGGGAAGCCAG
ATCCTGAAAGAGCACCCCGTGGAAAACACTCAGCTGCAGAATGAGAAGCTGTATCTGTACTATCTGCAGAATGGCAGGGATAT
GTACGTGGACCAGGAGCTGGATATTAACCGCCTGTCCGATTATGACGTGGATGCGATCGTCCCACAGTCATTCCTGAAAGAT
GACAGCATTGACAATAAGGTGCTGACCCGCTCTGACAAAAACCGAGGGAAGAGTGATAATGTCCCCTCAGAGGAAGTGGTCA
AGAAAATGAAGAACTACTGGCGGCAGCTGCTGAATGCCAAACTGATCACCCAGCGAAAGTTTGATAACCTGACAAAAGCTGA
GCGGGGAGGCCTGAGTGAACTGGACAAAGCAGGCTTCATTAAGCGACAGCTGGTGGAGACCCGGCAGATCACAAAGCACGT
CGCTCAGATTCTGGATAGCCGCATGAACACAAAGTACGATGAGAATGACAAACTGATCCGGGAAGTGAAGGTCATTACTCTGA
AGTCAAAACTGGTGAGCGACTTTCGGAAAGATTTCCAGTTTTATAAGGTCAGAGAGATCAACAACTACCACCATGCTCATGAC
GCATACCTGAACGCAGTGGTCGGAACCGCCCTGATTAAGAAATACCCTAAACTGGAGAGCGAATTCGTGTACGGCGACTATA
AGGTGTACGATGTCAGGAAAATGATCGCCAAGTCTGAGCAGGAAATTGGCAAAGCCACCGCTAAGTATTTCTTTTACAGTAAC
ATCATGAATTTCTTTAAGACTGAGATCACCCTGGCAAATGGCGAAATCCGAAAGCGGCCACTGATTGAGACTAACGGCGAGAC
CGGGGAAATCGTGTGGGACAAAGGGAGAGATTTTGCTACCGTGAGGAAGGTCCTGAGCATGCCCCAAGTGAATATTGTCAAG
AAAACAGAGGTGCAGACTGGGGGATTCAGTAAGGAATCAATTCTGCCTAAACGCAACTCCGATAAGCTGATCGCCCGAAAGA
AAGACTGGGATCCTAAGAAATATGGCGGGTTCGACTCCCCAACAGTGGCTTACTCTGTCCTGGTGGTCGCAAAGGTGGAGAA
GGGGAAAAGCAAGAAACTGAAATCCGTCAAGGAACTGCTGGGAATCACTATTATGGAGAGAAGCTCCTTCGAAAAGAATCCTA
TCGATTTTCTGGAGGCCAAAGGATATAAGGAAGTGAAGAAAGACCTGATCATCAAGCTGCCAAAGTACTCACTGTTTGAGCTG
GAAAACGGCAGAAAGAGGATGCTGGCAAGCGCCGGCGAGCTGCAGAAAGGGAATGAACTGGCCCTGCCCTCCAAGTACGTG
AACTTCCTGTATCTGGCTTCTCACTACGAGAAGCTGAAAGGCAGTCCTGAGGATAACGAACAGAAACAGCTGTTTGTGGAGCA
GCACAAGCATTATCTGGACGAGATCATTGAACAGATTAGCGAGTTCTCCAAAAGAGTGATCCTGGCTGACGCAAATCTGGATA
AGGTCCTGTCCGCATACAACAAACACCGGGATAAGCCAATCAGAGAGCAGGCCGAAAATATCATTCATCTGTTCACTCTGACC
AACCTGGGAGCACCAGCAGCCTTCAAGTATTTTGACACTACCATCGATCGCAAACGATACACATCTACTAAGGAGGTGCTGGA
CGCTACCCTGATCCACCAGAGTATTACTGGACTGTACGAGACCAGAATCGACCTGAGTCAGCTGGGAGGCGATTCAAGGGCC
GACCCTAAAAAGAAAAGAAAAGTCTGTACATATCCCTATGATGTCCCTGATTATGCCTAA

Supplementary Figure 6: Coding Sequence of SpCas9. The human optimized CDS of SpCas9 that we used in this study. The added NLS is in blue while the HA tag is in red.

Supplementary Figure 7

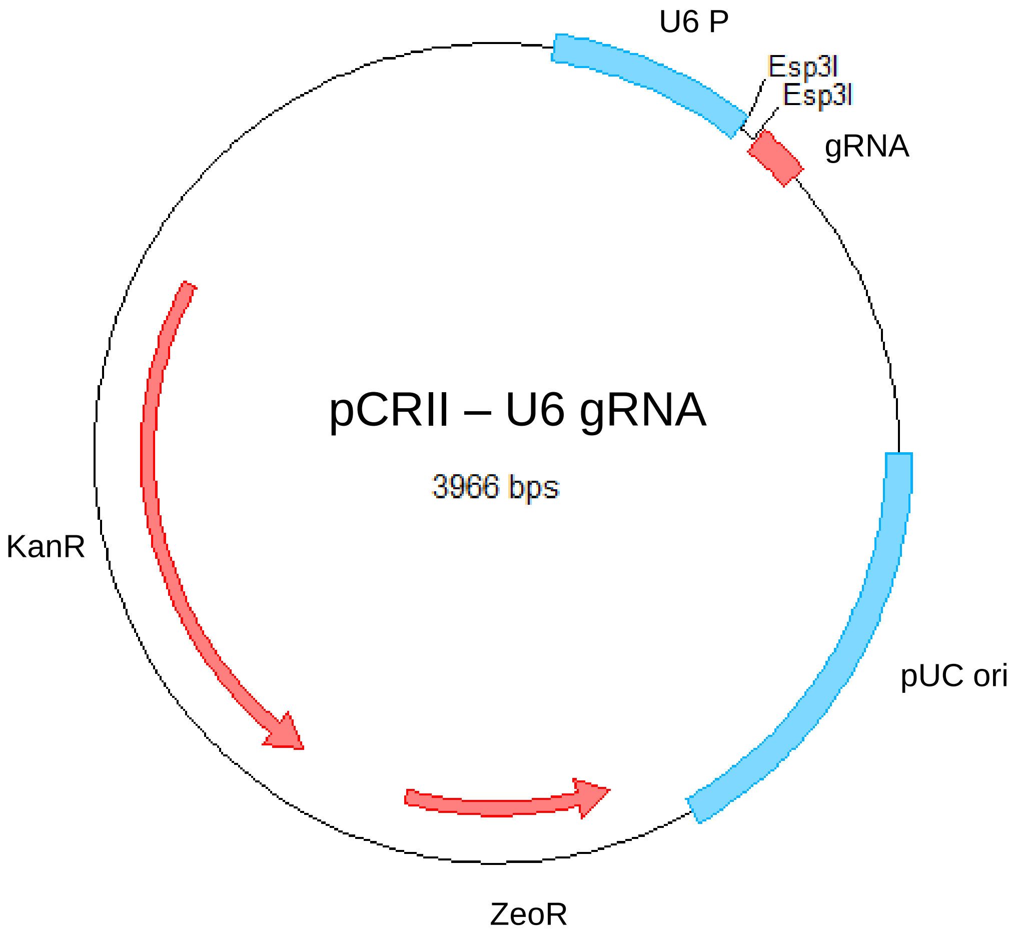

Supplementary Figure 7: pCRII – U6 gRNA Plasmid Map. The plasmid used to express gRNAs in mammalian cells drives gRNA expression with the U6 promoter. The cloning vector with a 34 bp insert was used to as a non-targeting control in gRNA transfections. Oligos were used to generate gRNAs by digesting the plasmid with Esp3I followed or in concert with ligation.

Supplementary Figure 8

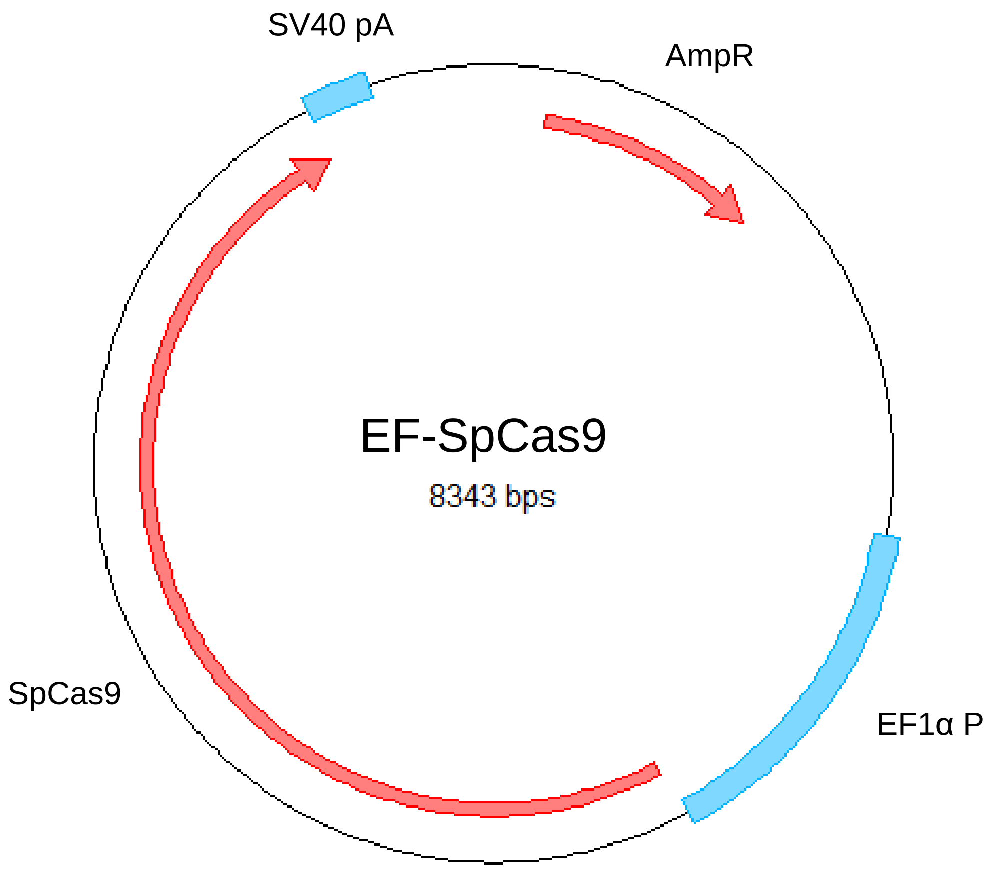

Supplementary Figure 8: EF-SpCas9 Plasmid Map. The plasmid used to express SpCas9 in mammalian cells drives SpCas9 expression with the EF1α promoter and terminates transcription with the SV40 pA signal.

Supplementary Figure 9

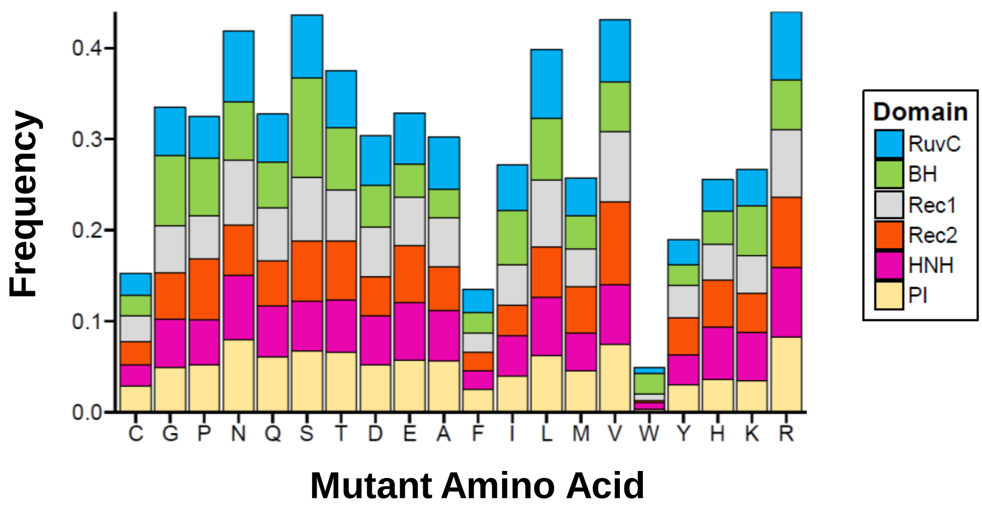

Supplementary Figure 9: Frequencies of specific amino acid substitutions by domain. Instances of amino acid substitutions from Fig. 2b were normalized by the total number of amino acids in each domain.

Supplementary Figure 10

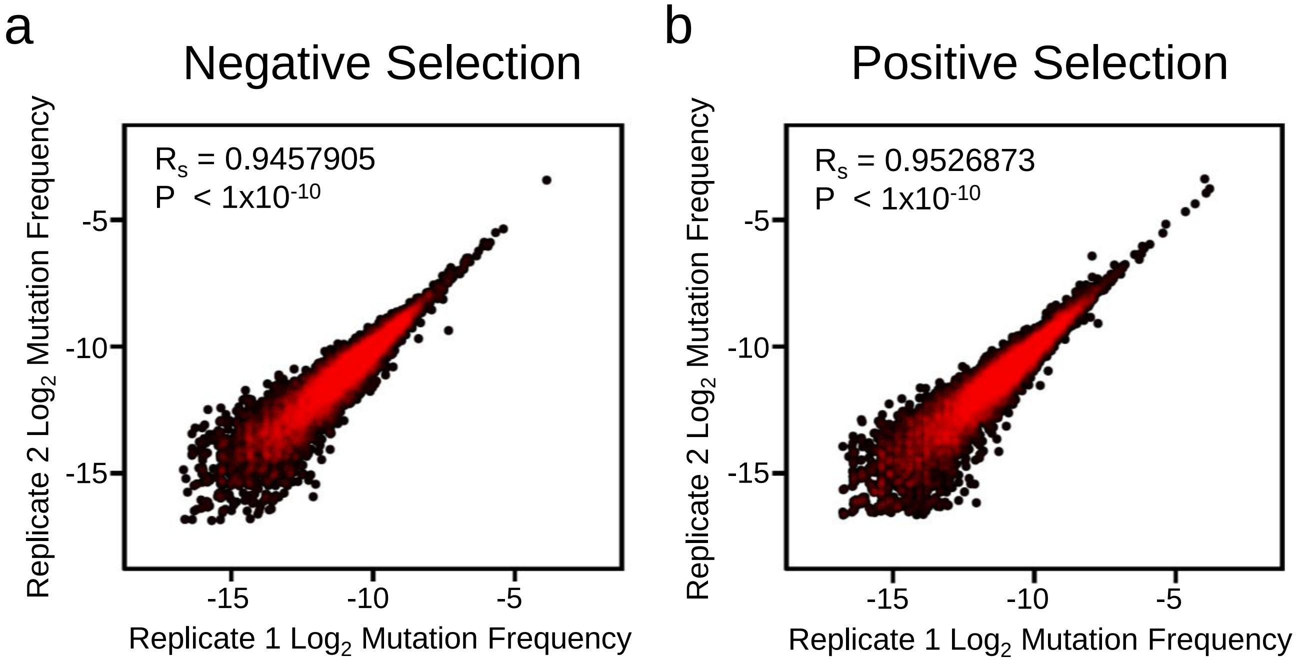

Supplementary Figure 10: Correlation of selection replicates. Selection replicates were converted to frequencies by adding one to the numerator and denominator of counts before calculating Spearman's Rho. Shown are representative plots of log_2 transformed frequencies and calculations from each selection.

Supplementary Figure 11

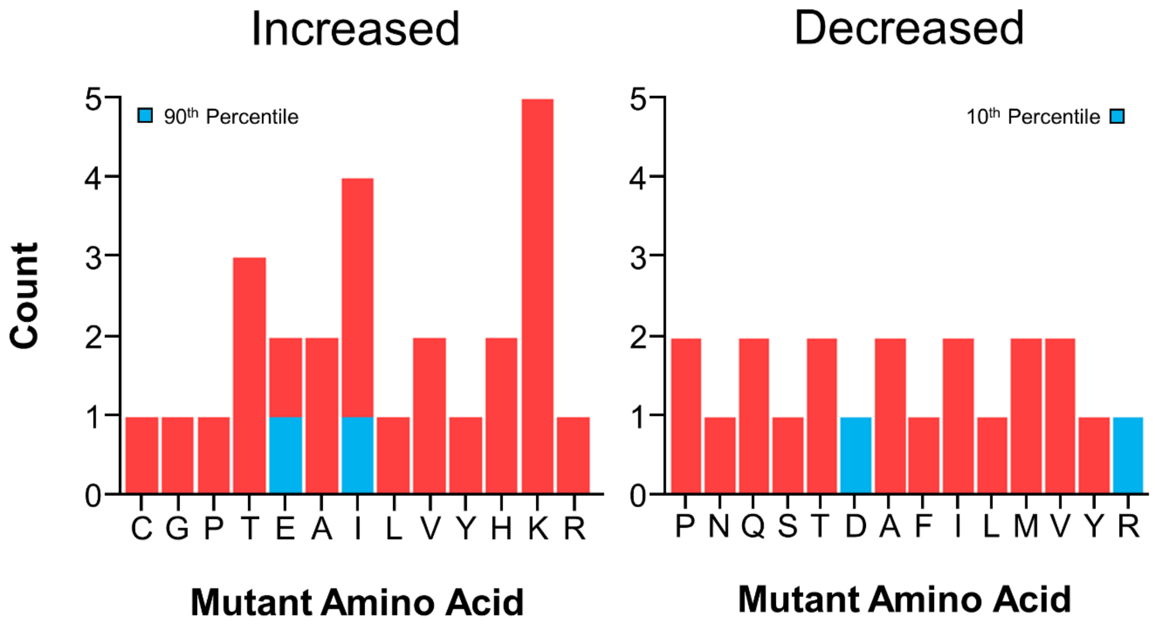

Supplementary Figure 11: Enriched and depleted mutations from negative selection. Histograms showing the number of enriched (Increased) or depleted (Decreased) amino acid mutations from negative selection. The top enriched and depleted mutations are colored in blue.

Supplementary Figure 12

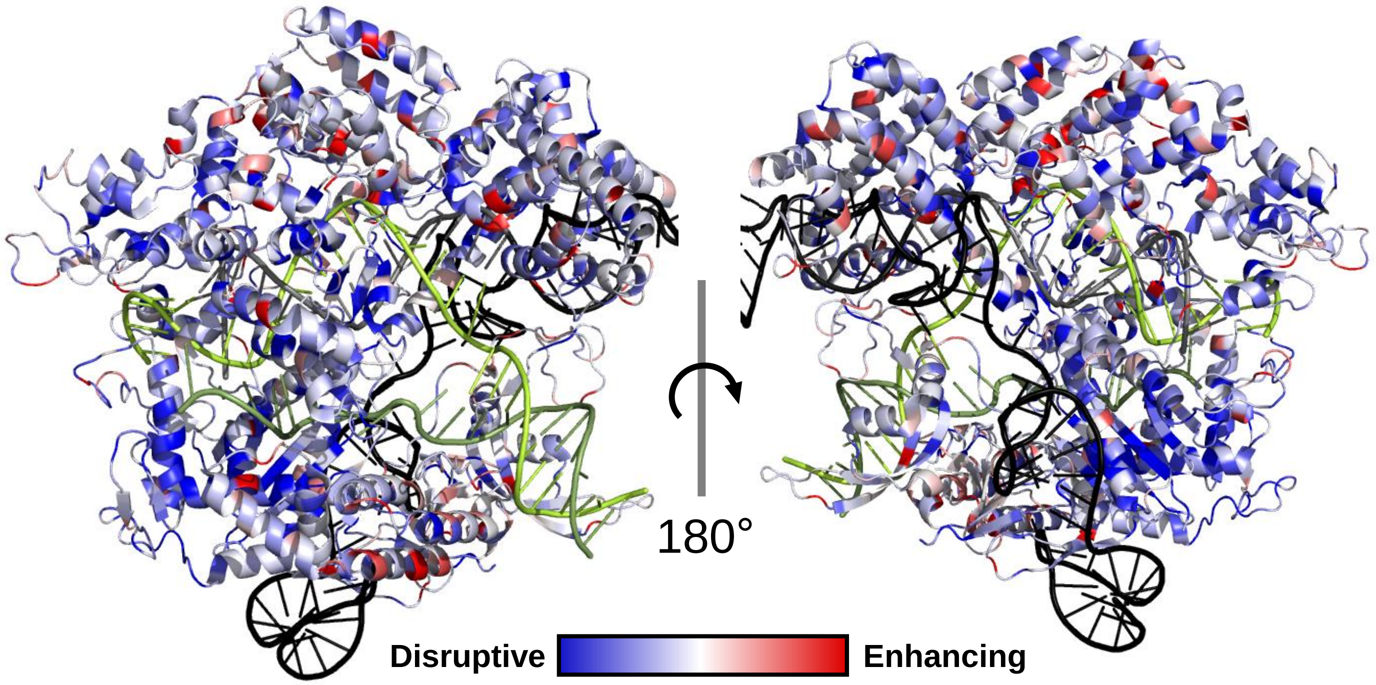

Supplementary Figure 12: Mutability score mapped onto the SpCas9 crystal structure. Mutability scores were calculated for each residue as explained in the methods. These scores were then mapped on the cartoon structure of SpCas9 bound to gRNA and dsDNA based on PDB: 5f9r (ref. 27). Red indicates residues with a mutability score that is greater than zero. Blue indicates a mutability score less than zero. White indicates a mutability score of zero. Color intensity is saturated at the top and bottom ten percentiles.

Supplementary Figure 13

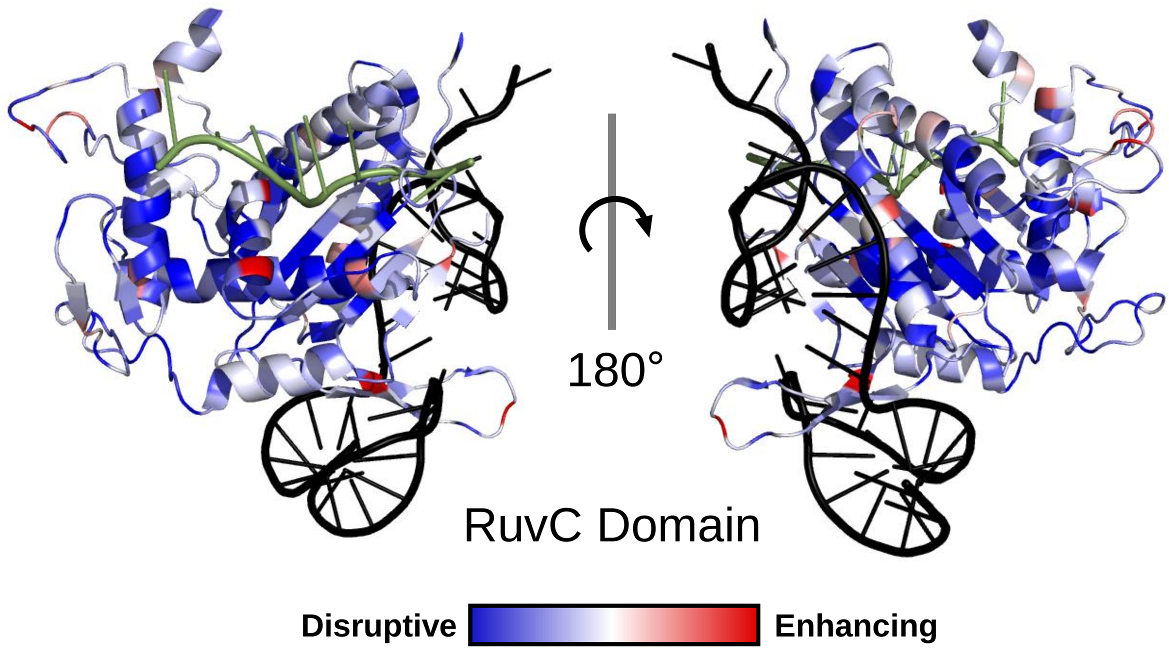

Supplementary Figure 13: Mutability score mapped onto the RuvC domain. A close-up view of the RuvC domain, colored as in Supplementary Fig. 12. Other domains have been removed for clarity.

Supplementary Figure 14

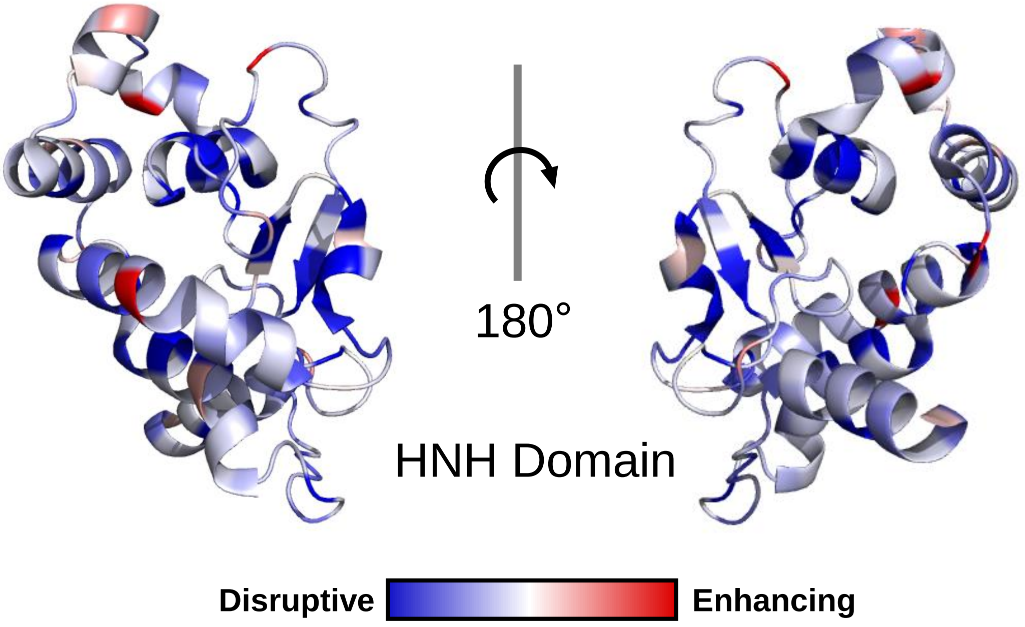

Supplementary Figure 14: Mutability score mapped onto the HNH domain. A close-up view of the HNH domain, colored as in Supplementary Fig. 12. Other domains have been removed for clarity.

Supplementary Figure 15

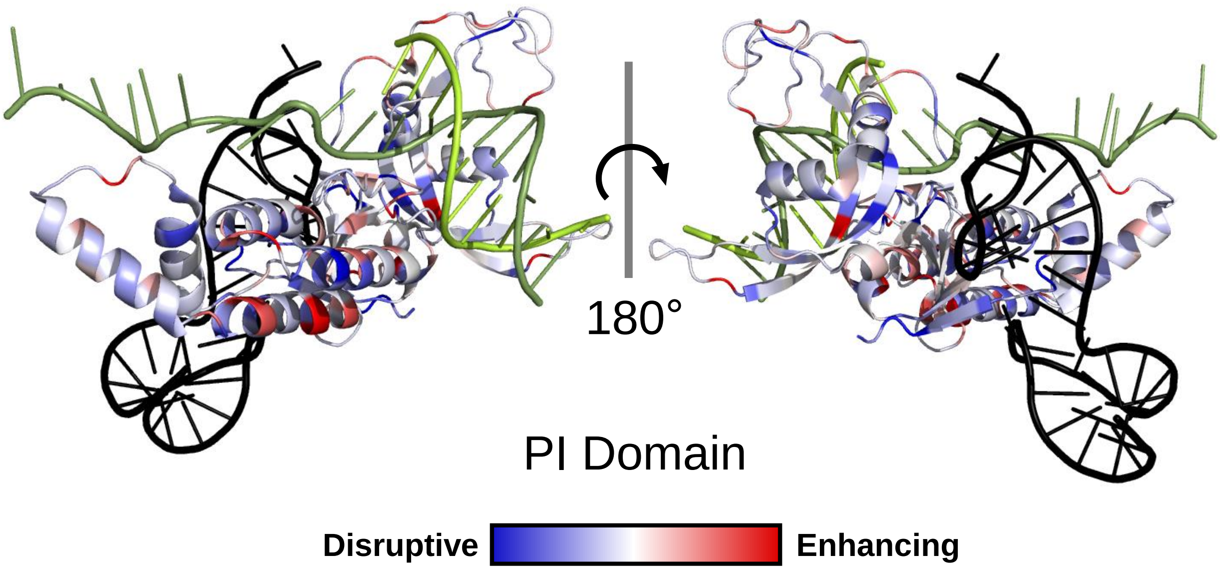

Supplementary Figure 15: Mutability score mapped onto the PI domain. A close-up view of the PI domain, colored as in Supplementary Fig. 12. Other domains have been removed for clarity.

Supplementary Figure 16

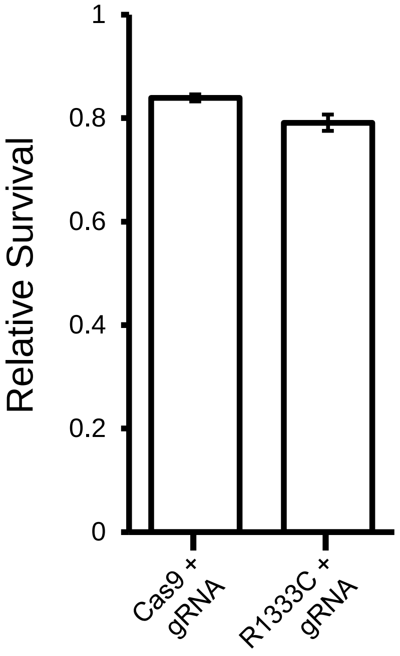

Supplementary Figure 16: Activity of R133C SpCas9 in *E. coli*. The positive selection plasmid was transformed along with an R1333C expression plasmid as in Figure 1 (n=3, error bars indicate S.E.M.). The results from the transformation are show alongside WT SpCas9 results from Figure 1c for comparison.

Supplementary Figure 17

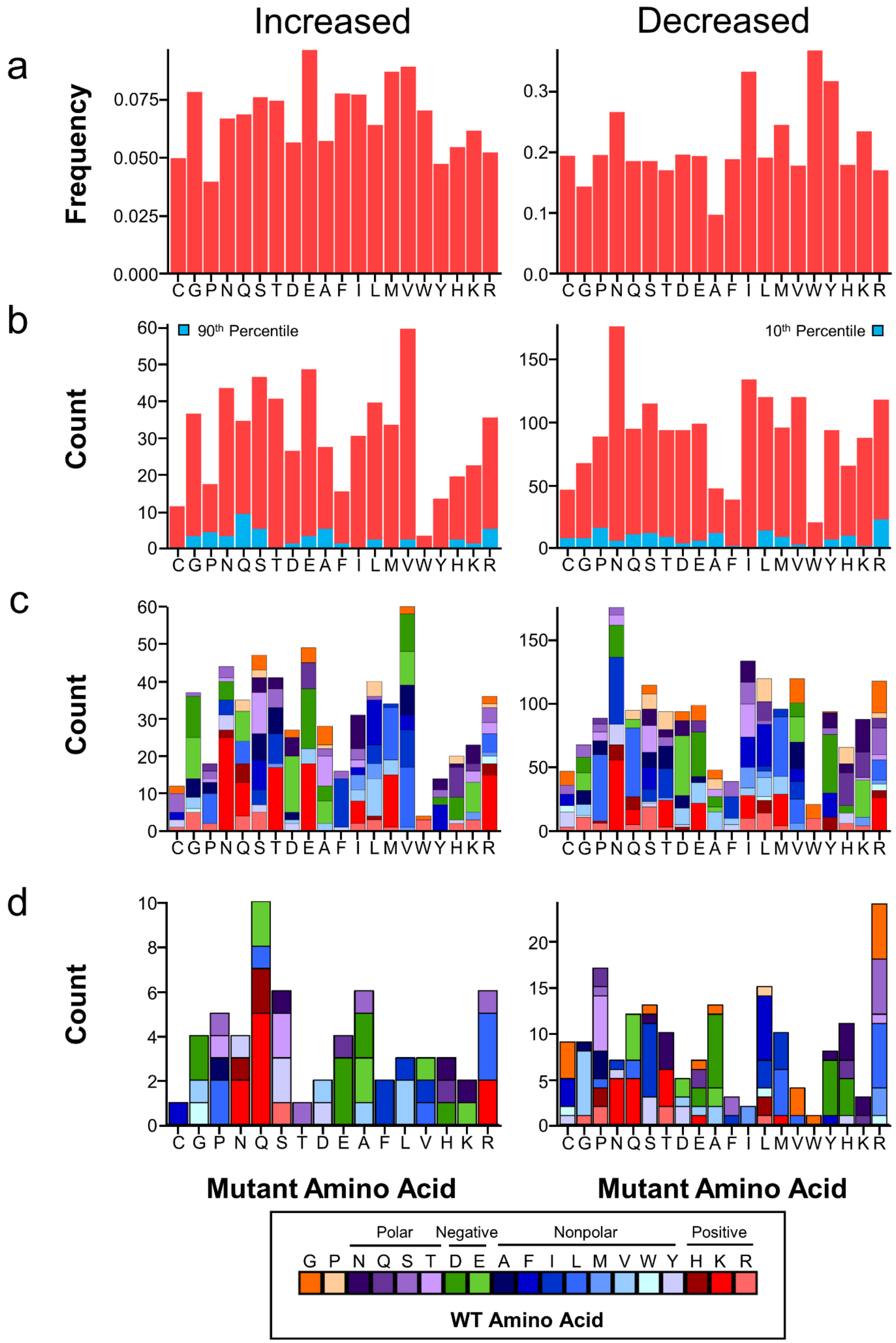

Supplementary Figure 17: Enriched and depleted mutations from positive selection. (a) Significantly enriched (Increased) or depleted (Decreased) mutation counts of each amino acid were divided by the total number instances of that mutation in the initial library. The frequency indicates the fraction of each amino acid mutation that was significantly enriched or depleted following positive selection. (b) Histograms showing the number of enriched or depleted amino acid mutations from positive selection. The top enriched and depleted mutations are colored in blue. (c) Same counts as in b, but colored by the WT amino acid that the mutation was substituting. (d) The top and bottom ten percent of enriched and depleted mutations colored as in c.

Supplementary Figure 18

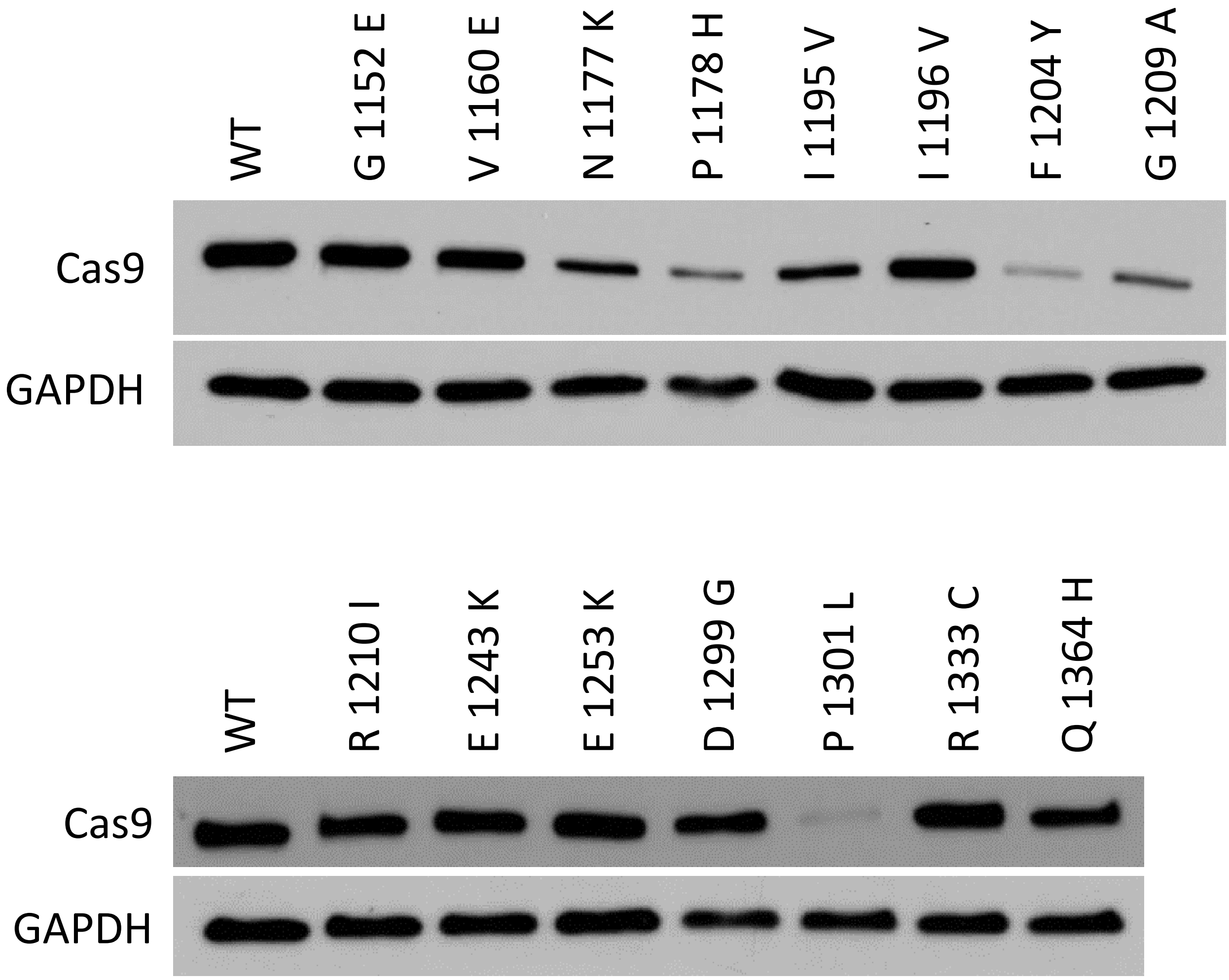

Supplementary Figure 18: Expression of specificity enhancing mutant Cas9s. Western blots of SpCas9 mutations from Fig. 4.

Supplementary Figure 19

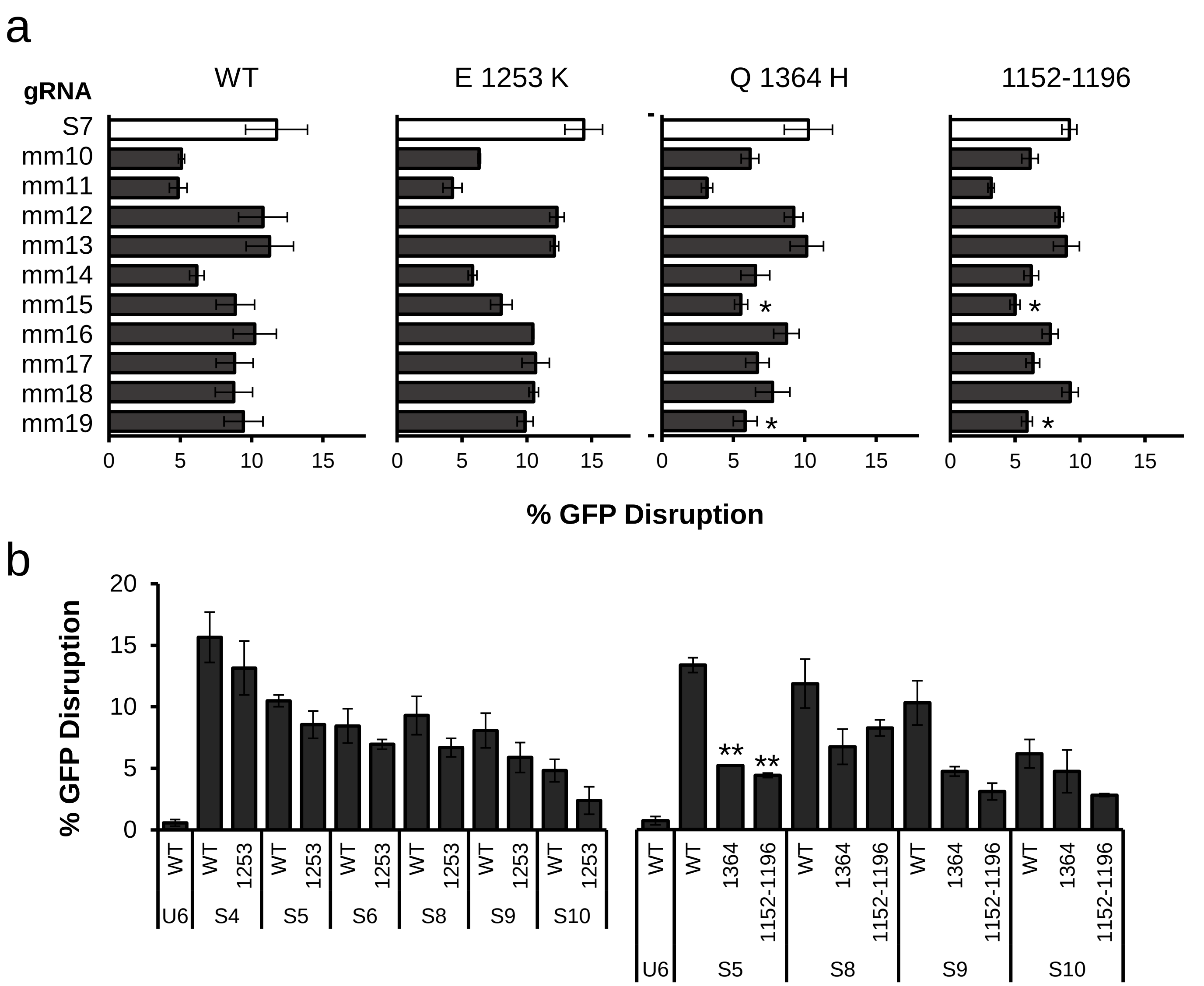

Supplementary Figure 19: General activity of doubly selected mutants. (a) Mutants from Figure 4 were assessed with an additional site 7 gRNA (S7) and their ability to tolerate single mismatches at the 5' end of the protospacer (n = 3, error bars indicate S.E.M.). Mismatches at the 15th (one-way ANOVA $F_{3,8}$ = 4.94, P = 0.032, followed by post hoc linear contrasts) and 19th position impaired function more than with WT for two of the mutants (one-way ANOVA $F_{3,8}$ = 6.14, P = 0.018, followed by post hoc linear contrasts). (b) These mutants were further assayed

with additional GFP-targeting gRNAs to determine their general on-target activity ($n = 2$, error bars indicate S.E.M., two-tailed Student's t-test, P = * < 0.05, ** < 0.01, *** < 0.001).

Supplementary Figure 20

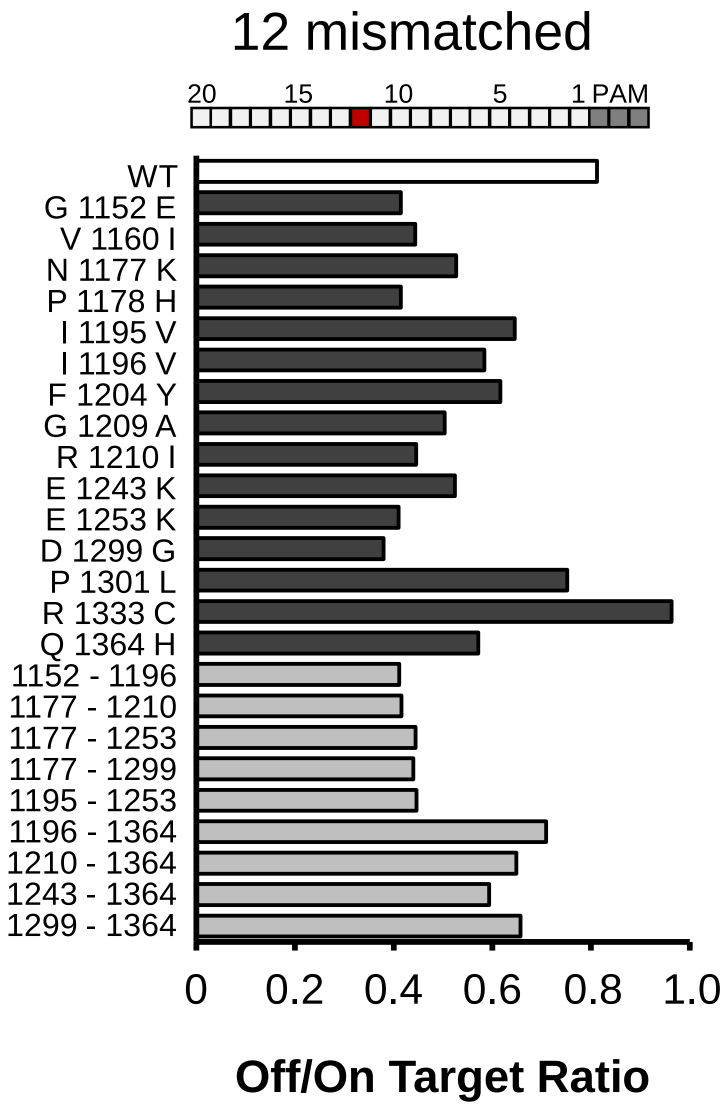

Supplementary Figure 20: Off/On Target Ratio of positive and negative selection enriched mutants. The off-target activity of each SpCas9 variant with the position 12 mismatched gRNA from Fig. 4 were divided by the on-target activity of each variant.

Supplementary Figure 21

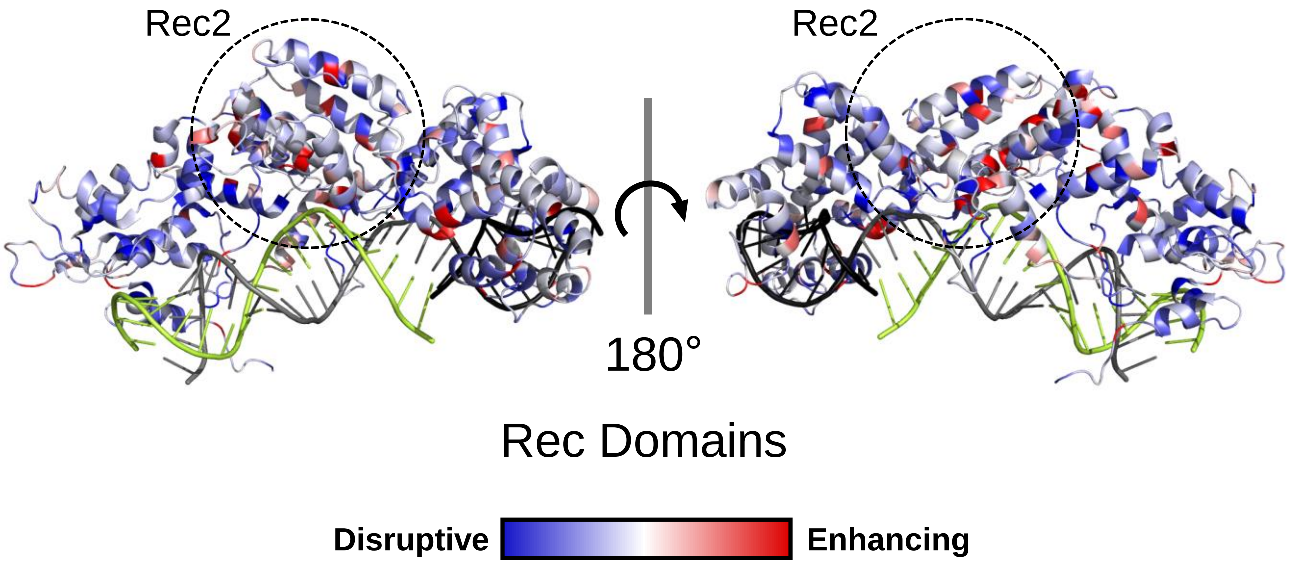

Supplementary Figure 21: Mutability score mapped onto the Rec domains. A close-up view of the Rec domains, colored as in Supplementary Fig. 12. Other domains have been removed for clarity. The Rec2 domain is enclosed in a dashed circle.

Supplementary Figure 22

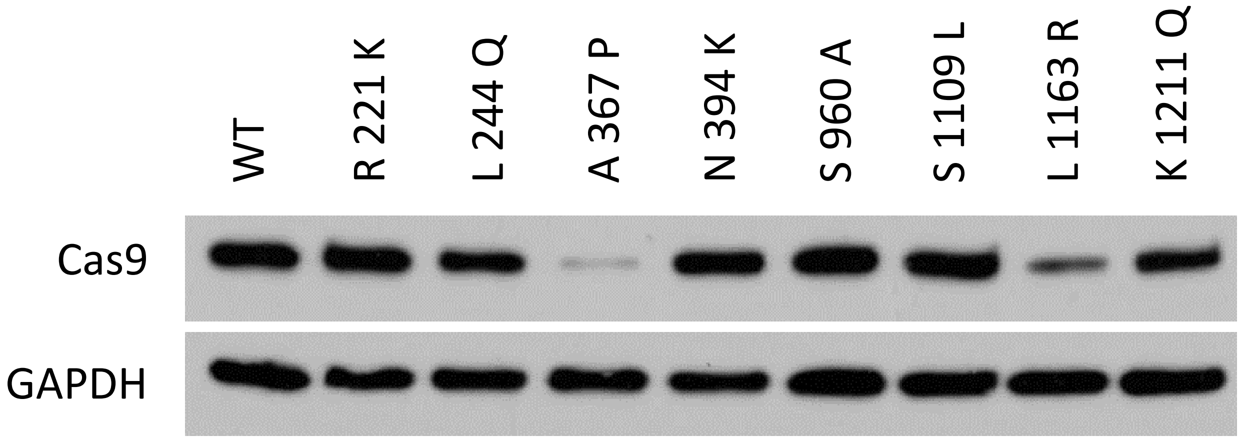

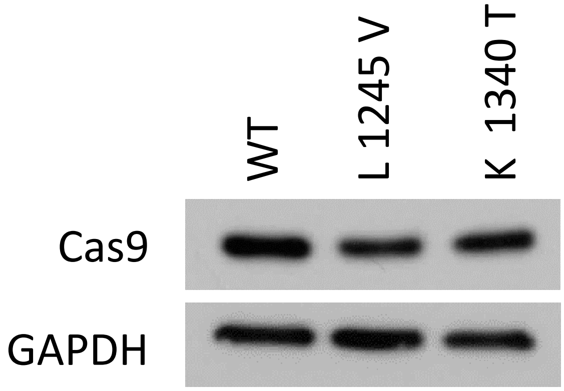

Supplementary Figure 22: Expression of enhancing mutation Cas9s. Western blots of SpCas9 mutations from Fig. 7.

Supplementary Figure 23

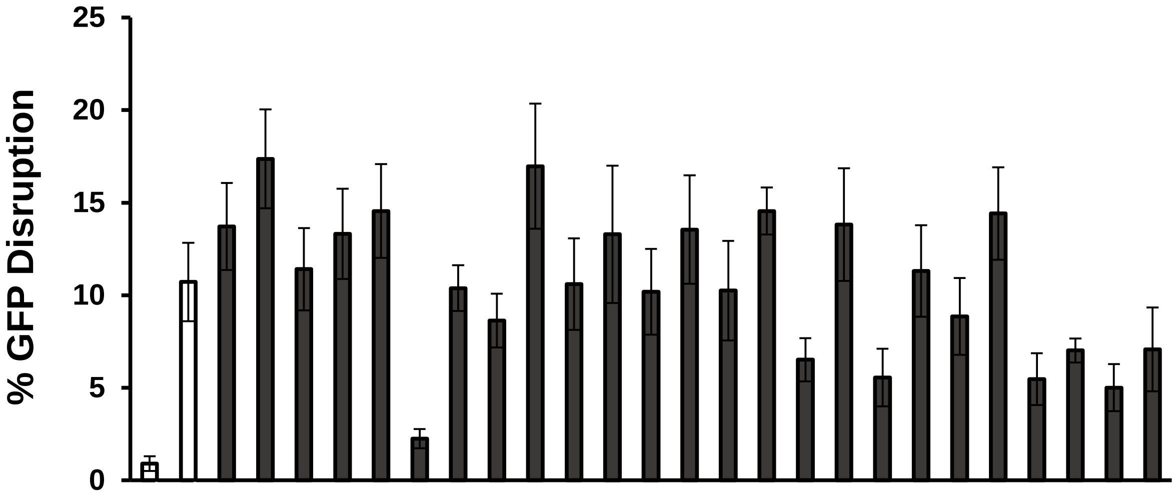

R 221 K	−	−	+	+	+	+	−	−	−	−	−	−	+	+	+	+	+	−	−	−	−	+	+	+	+	−	+
L 244 Q	−	−	+	−	−	−	+	+	+	−	−	−	+	+	+	−	−	+	+	+	−	+	+	+	−	+	+
N 394 K	−	−	−	+	−	−	+	−	−	+	+	−	+	−	−	+	+	+	+	−	+	+	+	−	+	+	+
K 1211 Q	−	−	−	−	+	−	−	+	−	+	−	+	−	+	−	+	−	+	−	+	+	+	−	+	+	+	+
L 1245 V	−	−	−	−	−	+	−	−	+	−	+	+	−	−	+	−	+	−	+	+	+	−	+	+	+	+	+
S7 gRNA	−	+	+	+	+	+	+	+	+	+	+	+	+	+	+	+	+	+	+	+	+	+	+	+	+	+	+

Supplementary Figure 23: Activity of combination mutants with site 7 gRNA. Combinations of mutations that had retained activity in mammalian cells as single mutants were compared to WT SpCas9's ability to cleave with a gRNA targeting GFP site 7 (S7) (n = 3, error bars indicate S.E.M.).

Supplementary Figure 24

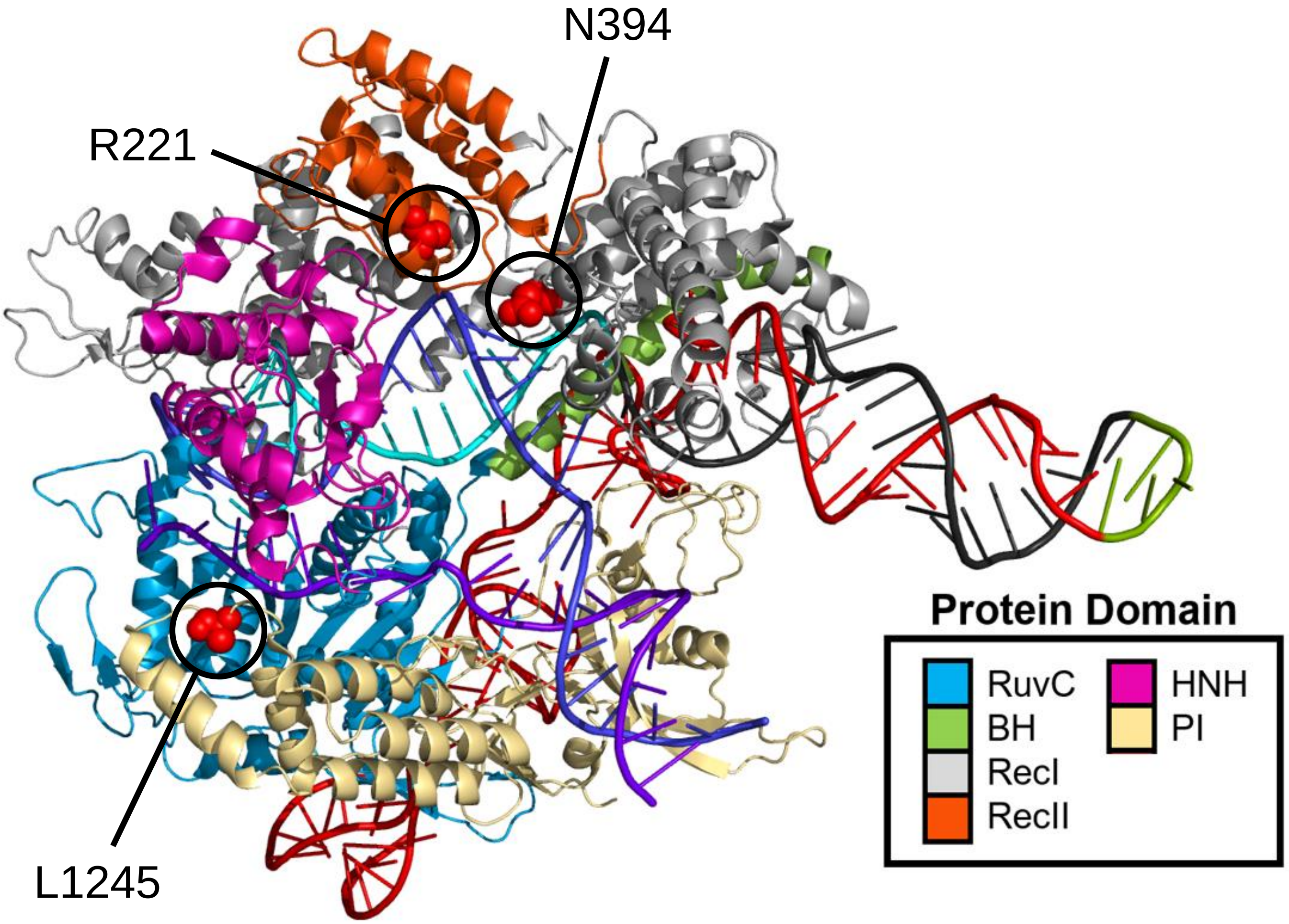

Supplementary Figure 24: Location of activating mutations. Cartoon structure of SpCas9 bound to gRNA and dsDNA based on PDB: 5f9r (ref. 27). Domains are colored as in Figure 4. The mutations which alter SpCas9 activity are shown as red spheres.

Supplementary Table 1: Primers used in this study

Name	Sequence (5' - 3')	Description
**pACYC184 -tacP- ccdB -T2 Construction**		
tacP For	CCGAGCTGTTGACAATTAATCATCGGCTGTATAATGTGTGGAATTGTGAGCGGATAACAATTTCA	tacP ccdB amplicon primer
ccdb Rev	GTTTAAACTTCATCCGGGGTCAGCACCGTTTCT	tacP ccdB amplicon primer
**Mutagenesis**		
EF For	CACACTGAGTGGGTGGAGACTG	Mutagenesis Primer
Sv40 For	TTAATTAAAACTTGTTTATTGCAGCTTATAATG	Mutagenesis Primer
**EF- RBS-SpCas9 Construction**		
SV40 POE For	GCCTAACGTGAGACGAAGGATGCGGCCGCGACATGATAAGATACATTGA	POE-PCR Vector Primer
EF- POE - Rev	TGTGTGAAGAGACGAGAGAACTGGGTGGCGCTAGTGAATTCCTCACGAC	POE-PCR Vector Primer
Cas9 RBS For 4	CAGTTCTCTCGTCTCTTCACACAGGAAACCTCATAGATGGATAAAAAATACTCAATCGGGCTGGA	POE-PCR Cas9 amplicon Primer
HA Rev 4	CATCCTTCGTCTCACGTTAGGCATAATCAGGGACATCATAGGGA	POE-PCR Cas9 amplicon Primer
**Mutation Cloning Primers**		
G-Cas For	AAAATACTCAATCGGGCTGGACATCGGGA	N-terminal Fragment Primer
G-Cas Rev	CGCTTAGGCATAATCAGGGACATCATAGGGA	C-terminal Fragment Primer
1152-E For	GGTCGCAAAGGTGGAGAAGGAGAAAAGCAAGAAACTGAAATC	1152-E Cloning
1152-E Rev	GATTTCAGTTTCTTGCTTTTCTCCTTCTCCACCTTTGCGACC	1152-E Cloning
1160-I For	AAAGCAAGAAACTGAAATCCATCAAGGAACTGCTGGGAATC	1160-I Cloning
1160-I Rev	GATTCCCAGCAGTTCCTTGATGGATTTCAGTTTCTTGCTTT	1160-I Cloning
1177-K For	AGAAGCTCCTTCGAAAAGAAACCTATCGATTTTCTGGAGGC	1177-K Cloning
1177-K Rev	GCCTCCAGAAAATCGATAGGTTTCTTTTCGAAGGAGCTTCT	1177-K Cloning
1178-H For	AAGCTCCTTCGAAAAGAATCATATCGATTTTCTGGAGGCCAAAG	1178-H Cloning
1178-H Rev	CTTTGGCCTCCAGAAAATCGATATGATTCTTTTCGAAGGAGCTT	1178-H Cloning
1195-V For	AGGAAGTGAAGAAAGACCTGGTCATCAAGCTGCCAAAGTAC	1195-V Cloning
1195-V Rev	GTACTTTGGCAGCTTGATGACCAGGTCTTTCTTCACTTCCT	1195-V Cloning
1196-V For	AAGTGAAGAAAGACCTGATC**G**TCAAGCTGCCAAAGTACTCAC	1196-V Cloning
1196-V Rev	GTGAGTACTTTGGCAGCTTGACGATCAGGTCTTTCTTCACTT	1196-V Cloning
1204-Y For	AGCTGCCAAAGTACTCACTGTATGAGCTGGAAAACGGCAGAAAG	1204-Y Cloning
1204-Y Rev	CTTTCTGCCGTTTTCCAGCTCATACAGTGAGTACTTTGGCAGCT	1204-Y Cloning
1209-A For	ACTGTTTGAGCTGGAAAACG**C**CAGAAAGAGGATGCTGGCAAG	1209-A Cloning
1209-A Rev	CTTGCCAGCATCCTCTTTCTGGCGTTTTCCAGCTCAAACAGT	1209-A Cloning
1210-I For	GTTTGAGCTGGAAAACGGCA**T**AAAGAGGATGCTGGCAAGCG	1210-I Cloning
1210-I Rev	CGCTTGCCAGCATCCTCTTTATGCCGTTTTCCAGCTCAAAC	1210-I Cloning

1243-K For	TGTATCTGGCTTCTCACTACAAGAAGCTGAAAGGCAGTCCTG	1243-K Cloning
1243-K Rev	CAGGACTGCCTTTCAGCTTCTTGTAGTGAGAAGCCAGATACA	1243-K Cloning
1253-K For	AAGGCAGTCCTGAGGATAACAAACAGAAACAGCTGTTTGTG	1253-K Cloning
1253-K Rev	CACAAACAGCTGTTTCTGTTTGTTATCCTCAGGACTGCCTT	1253-K Cloning
1299-G For	CGCATACAACAAACACCGGGGTAAGCCAATCAGAGAGCAGG	1299-G Cloning
1299-G Rev	CCTGCTCTCTGATTGGCTTACCCCGGTGTTTGTTGTATGCG	1299-G Cloning
1301-L For	CATACAACAAACACCGGGATAAGCTAATCAGAGAGCAGGCCGAAAA TATC	1301-L Cloning
1301-L Rev	GATATTTTCGGCCTGCTCTCTGATTAGCTTATCCCGGTGTTTGTTGT ATG	1301-L Cloning
1333-C For	GTATTTTGACACTACCATCGATTGCAAACGATACACATCTACTAAG	1333-C Cloning
1333-C Rev	CTTAGTAGATGTGTATCGTTTGCAATCGATGGTAGTGTCAAAATAC	1333-C Cloning
1364-H For	CGAGACCAGAATCGACCTGAGTCACCTGGGAGGCGATTCAAGGGC C	1364-H Cloning
1364-H Rev	GGCCCTTGAATCGCCTCCCAGGTGACTCAGGTCGATTCTGGTCTCG	1364-H Cloning
221-K For	CCGCCTGTCTAAGAGTCGGAAACTGGAGAACCTGATCGCT	221-K Cloning
221-K Rev	AGCGATCAGGTTCTCCAGTTTCCGACTCTTAGACAGGCGG	221-K Cloning
244-Q For	GGACTGTTTGGCAATCTGATTGCACAGTCCCTGGGGCTGACACCCA AC	244-Q Cloning
244-Q Rev	GTTGGGTGTCAGCCCCAGGGACTGTGCAATCAGATTGCCAAACAGT CC	244-Q Cloning
367-P For	CCGGATATATTGACGGCGGGCCTTCCCAGGAGGAATTCTACAAG	367-P Cloning
367-P Rev	CTTGTAGAATTCCTCCTGGGAAGGCCCGCCGTCAATATATCCGG	367-P Cloning
394-K For	AGGAACTGCTGGTGAAACTGAAGAGGGAAGACCTGCTGAGGAAGC A	394-K Cloning
394-K Rev	TGCTTCCTCAGCAGGTCTTCCCTCTTCAGTTTCACCAGCAGTTCCT	394-K Cloning
960-A For	GGAAGTGAAGGTCATTACTCTGAAGGCAAAACTGGTGAGCGACTTT CGG	960-A Cloning
960-A Rev	CCGAAAGTCGCTCACCAGTTTTGCCTTCAGAGTAATGACCTTCACTT CC	960-A Cloning
1109-L For	GGATTCAGTAAGGAATTAATTCTGCCTAAACGCAACTCCGATAAG	1109-L Cloning
1109-L Rev	CTTATCGGAGTTGCGTTTAGGCAGAATTAATTCCTTACTGAATCC	1109-L Cloning
1163-R For	AGAAACTGAAATCCGTCAAGGAACGGCTGGGAATCACTATTATGGA GAG	1163-R Cloning
1163-R Rev	CTCTCCATAATAGTGATTCCCAGCCGTTCCTTGACGGATTTCAGTTT CT	1163-R Cloning
1211-Q For	GCCTGACCTTCAAAGAGGATATCCAGAAGGCAGAGGTGTCCGGCC AGG	1211-Q Cloning
1211-Q Rev	CCTGGCCGGACACCTCTGCCTTCTGGATATCCTCTTTGAAGGTCAG GC	1211-Q Cloning
1245-V For	ATCTGGCTTCTCACTACGAGAAGGTGAAAGGCAGTCCTGAGGATAA CG	1245-V Cloning
1245-V Rev	CGTTATCCTCAGGACTGCCTTTCACCTTCTCGTAGTGAGAAGCCAG AT	1245-V Cloning
1340-T For	GATCGCAAACGATACACATCTACTACGGAGGTGCTGGACGCTACCC TG	1340-T Cloning
1340-T Rev	CAGGGTAGCGTCCAGCACCTCCGTAGTAGATGTGTATCGTTTGCGA TC	1340-T Cloning

NGS Primers		
ProD For	GGGCATGCATAAGGCTCGTATAATA	Initial Sequencing Amplicon
Omega Rev2	GCAGCGCGATTACAGTGGTTT	Initial Sequencing Amplicon
Spfor-3	TCGTCGGCAGCGTCAGATGTGTATAAGAGACAGNNNNNNNNagctcacacaggaaacctca	Fragment 1 Amplicon
Sprev214	GTCTCGTGGGCTCGGAGATGTGTATAAGAGACAGNNNNNNNNtccgattctttcggcgagta	Fragment 1 Amplicon
Spfor215	TCGTCGGCAGCGTCAGATGTGTATAAGAGACAGNNNNNNNNagaacagctcggagaaggta	Fragment 2 Amplicon
Sprev442	GTCTCGTGGGCTCGGAGATGTGTATAAGAGACAGNNNNNNNNagatcagtctcaggtctgct	Fragment 2 Amplicon
Spfor441	TCGTCGGCAGCGTCAGATGTGTATAAGAGACAGNNNNNNNNgaaactggtggactctaccgataa	Fragment 3 Amplicon
Sprev652	GTCTCGTGGGCTCGGAGATGTGTATAAGAGACAGNNNNNNNNggttctccagtctccgactc	Fragment 3 Amplicon
Spfor653	TCGTCGGCAGCGTCAGATGTGTATAAGAGACAGNNNNNNNNtgagcgcccgcctgtctaa	Fragment 4 Amplicon
Sprev865	GTCTCGTGGGCTCGGAGATGTGTATAAGAGACAGNNNNNNNNgattcttagcggccaggaac	Fragment 4 Amplicon
Spfor866	TCGTCGGCAGCGTCAGATGTGTATAAGAGACAGNNNNNNNNggcgatcagtacgcagacct	Fragment 5 Amplicon
Sprev1078	GTCTCGTGGGCTCGGAGATGTGTATAAGAGACAGNNNNNNNNccccgccgtcaatatatccg	Fragment 5 Amplicon
Spfor1079	TCGTCGGCAGCGTCAGATGTGTATAAGAGACAGNNNNNNNNtcagagcaagaatgggtacgc	Fragment 6 Amplicon
Sprev1269	GTCTCGTGGGCTCGGAGATGTGTATAAGAGACAGNNNNNNNNtgggtagaagtcttcctggc	Fragment 6 Amplicon
Spfor1270	TCGTCGGCAGCGTCAGATGTGTATAAGAGACAGNNNNNNNNctgcacgccatcctgaggc	Fragment 7 Amplicon
Sprev1490	GTCTCGTGGGCTCGGAGATGTGTATAAGAGACAGNNNNNNNNgtttggcaggttcttgtcgaa	Fragment 7 Amplicon
Spfor1491	TCGTCGGCAGCGTCAGATGTGTATAAGAGACAGNNNNNNNNagtctttcattgagaggatgacaaac	Fragment 8 Amplicon
Sprev1678	GTCTCGTGGGCTCGGAGATGTGTATAAGAGACAGNNNNNNNNtcctctttcagctgcttgac	Fragment 8 Amplicon
Spfor1679	TCGTCGGCAGCGTCAGATGTGTATAAGAGACAGNNNNNNNNgtttaaaaccaatcggaaggtgac	Fragment 9 Amplicon
Sprev1895	GTCTCGTGGGCTCGGAGATGTGTATAAGAGACAGNNNNNNNNcataagtcttcaggcgttcct	Fragment 9 Amplicon
Spfor1896	TCGTCGGCAGCGTCAGATGTGTATAAGAGACAGNNNNNNNNctgtttgaggatagggaaatgatc	Fragment 10 Amplicon
Sprev2118	GTCTCGTGGGCTCGGAGATGTGTATAAGAGACAGNNNNNNNNcacctgtgccttctggatat	Fragment 10 Amplicon

Spfor2119	TCGTCGGCAGCGTCAGATGTGTATAAGAGACAGNNNNNNNNc agcctgaccttcaaagagg	Fragment 11 Amplicon
Sprev2342	GTCTCGTGGGCTCGGAGATGTGTATAAGAGACAGNNNNNNNN atcccttcctcgatgcgttt	Fragment 11 Amplicon
Spfor2343	TCGTCGGCAGCGTCAGATGTGTATAAGAGACAGNNNNNNNNa gaactcaagggagcgcatg	Fragment 12 Amplicon
Sprev2570	GTCTCGTGGGCTCGGAGATGTGTATAAGAGACAGNNNNNNNN cggtttttgtcagagcgggt	Fragment 12 Amplicon
Spfor2571	TCGTCGGCAGCGTCAGATGTGTATAAGAGACAGNNNNNNNNc agcattgacaataaggtgctg	Fragment 13 Amplicon
Sprev2794	GTCTCGTGGGCTCGGAGATGTGTATAAGAGACAGNNNNNNNN atgcggctatccagaatctg	Fragment 13 Amplicon
Spfor2795	TCGTCGGCAGCGTCAGATGTGTATAAGAGACAGNNNNNNNNa gatcacaaagcacgtcgct	Fragment 14 Amplicon
Sprev3020	GTCTCGTGGGCTCGGAGATGTGTATAAGAGACAGNNNNNNNN ccttatagtcgccgtacacgaa	Fragment 14 Amplicon
Spfor3021	TCGTCGGCAGCGTCAGATGTGTATAAGAGACAGNNNNNNNNa ccctaaactggagagcgaa	Fragment 15 Amplicon
Sprev3217	GTCTCGTGGGCTCGGAGATGTGTATAAGAGACAGNNNNNNNN aaatctctccctttgtcccac	Fragment 15 Amplicon
Spfor3218	TCGTCGGCAGCGTCAGATGTGTATAAGAGACAGNNNNNNNNg cgagaccggggaaatcgt	Fragment 16 Amplicon
Sprev3428	GTCTCGTGGGCTCGGAGATGTGTATAAGAGACAGNNNNNNNN tccacctttgcgaccaccag	Fragment 16 Amplicon
Spfor3429	TCGTCGGCAGCGTCAGATGTGTATAAGAGACAGNNNNNNNNc caacagtggcttactctgtc	Fragment 17 Amplicon
Sprev3632	GTCTCGTGGGCTCGGAGATGTGTATAAGAGACAGNNNNNNNN ggcgcttgccagcatcct	Fragment 17 Amplicon
Spfor3633	TCGTCGGCAGCGTCAGATGTGTATAAGAGACAGNNNNNNNNa gctggaaaacggcagaaag	Fragment 18 Amplicon
Sprev3852	GTCTCGTGGGCTCGGAGATGTGTATAAGAGACAGNNNNNNNN ggacaggaccttatccagatttg	Fragment 18 Amplicon
Spfor3853	TCGTCGGCAGCGTCAGATGTGTATAAGAGACAGNNNNNNNNa agagtgatcctggctgacg	Fragment 19 Amplicon
Sprev4070	GTCTCGTGGGCTCGGAGATGTGTATAAGAGACAGNNNNNNNN tgactcaggtcgattctggt	Fragment 19 Amplicon
Spfor4071	TCGTCGGCAGCGTCAGATGTGTATAAGAGACAGNNNNNNNNa gagtattactggactgtacgag	Fragment 20 Amplicon
Sprev4246	GTCTCGTGGGCTCGGAGATGTGTATAAGAGACAGNNNNNNNN cgcctacctgcatctgacga	Fragment 20 Amplicon

Supplementary Table 2: Plasmids used in this study

Plasmid Name	Description	Source
pUC-ProD-Cas9-T2	Cas9 and T2 gRNA bacterial expression plasmid	This work
pUC-ProD-LacZ-T2	Cloning plasmid used to generate mutant library	This work
EF-SpCas9	Cas9 mammalian expression plasmid	Our lab
EF- RBS-SpCas9	Template plasmid for ep-PCR	This work
pCRII – U6 gRNA	gRNA mammalian expression plasmid	Our lab
pACYC184 -tacP-ccdB -T2	Positive selection plasmid	This work
pH3 – OT9	Negative selection plasmid	This work
pACYC184	Parent vector of positive selection plasmid	NEB
pH3U3-mcs	Parent vector of negative selection plasmid	Addgene plasmid # 12609

Supplementary Table 3: Oligos used in this study

Sense Name	Sense Oligo	Anti-Sense Name	Anti-sense Oligo
**GFP Targeting gRNAs**			
Site1 (s)	ACCGGGCACGGGCAGCTTGCCGG	Site1 (a)	AAACCCGGCAAGCTGCCCGTGCC
Site 4 (s)	ACCGGCGAGGGCGATGCCACCTA	Site 4 (a)	AAACTAGGTGGCATCGCCCTCGC
Site 5 (s)	ACCGGTCGCCACCATGGTGAGCA	Site 5 (a)	AAACTGCTCACCATGGTGGCGAC
Site 6 (s)	ACCGGTCAGGGTGGTCACGAGGG	Site 6 (a)	AAACCCCTCGTGACCACCCTGAC
Site 7 (s)	ACCGGTGGTGCAGATGAACTTCA	Site 7 (a)	AAACTGAAGTTCATCTGCACCAC
Site 8 (s)	ACCGTTGGGGTCTTTGCTCAGGG	Site 8 (a)	AAACCCCTGAGCAAAGACCCCAA
Site 9 (s)	ACCGGTGGTCACGAGGGTGGGCC	Site 9 (a)	AAACGGCCCACCCTCGTGACCAC
Site 10 (s)	ACCGATGCCGTTCTTCTGCTTGT	Site 10 (a)	AAACACAAGCAGAAGAACGGCAT
Site 1 mm 12 (s)	ACCGGGCACGGCCAGCTTGCCGG	Site 1 mm 12 (a)	AAACCCGGCAAGCTGGCCGTGCC
Site 1 mm 19 & 18 (s)	ACCGCCCACGGGCAGCTTGCCGG	Site 1 mm 19 & 18 (a)	AAACCCGGCAAGCTGCCCGTGGG
Site 7 mm 19 (s)	ACCGCTGGTGCAGATGAACTTCA	Site 7 mm 19 (a)	AAACTGAAGTTCATCTGCACCAG
Site 7 mm 18 (s)	ACCGGAGGTGCAGATGAACTTCA	Site 7 mm 18 (a)	AAACTGAAGTTCATCTGCACCTC
Site 7 mm 17 (s)	ACCGGTCGTGCAGATGAACTTCA	Site 7 mm 17 (a)	AAACTGAAGTTCATCTGCACGAC
Site 7 mm 16 (s)	ACCGGTGCTGCAGATGAACTTCA	Site 7 mm 16 (a)	AAACTGAAGTTCATCTGCAGCAC
Site 7 mm 15 (s)	ACCGGTGGAGCAGATGAACTTCA	Site 7 mm 15 (a)	AAACTGAAGTTCATCTGCTCCAC
Site 7 mm 14 (s)	ACCGGTGGTCCAGATGAACTTCA	Site 7 mm 14 (a)	AAACTGAAGTTCATCTGGACCAC
Site 7 mm 13 (s)	ACCGGTGGTGGAGATGAACTTCA	Site 7 mm 13 (a)	AAACTGAAGTTCATCTCCACCAC
Site 7 mm 12 (s)	ACCGGTGGTGCTGATGAACTTCA	Site 7 mm 12 (a)	AAACTGAAGTTCATCAGCACCAC
Site 7 mm 11 (s)	ACCGGTGGTGCACATGAACTTCA	Site 7 mm 11 (a)	AAACTGAAGTTCATGTGCACCAC
Site 7 mm 10 (s)	ACCGGTGGTGCAGTTGAACTTCA	Site 7 mm 10 (a)	AAACTGAAGTTCAACTGCACCAC
**pH3 Targets**			
2x - OT9 (s)	GAGCTCGCCCCCACCCACCCCGCCTCCGGAGCCCCCACCCACCCCGCCTCCGG	2x - OT9 (a)	CCGGCCGGAGGCGGGGTGGGTGGGGGCTCCGGAGGCGGGGTGGGTGGGGGCGAGCTCGACGT
**pACYC184 Targets**			
4x - T2 (s)	TCGACGAATTCACTAAGACCCCCTCCACCCCGCCTCCGGGACCCCCTCCACCCCGCCTCCGGGACCCCCTCCACCCCGCCTCCGGGACCCCCTCCACCCCGCCTCCGG	4x -T2 (a)	TCGACCGGAGGCGGGGTGGAGGGGGTCCCGGAGGCGGGGTGGAGGGGGTCCCGGAGGCGGGGTGGAGGGGGTCCCGGAGGCGGGGTGGAGGGGGTCTTAGTGAATTCG
1x - T2 (s)	TCGGGAATTCGACCCCCTCCACCCCGCCTCCGG	1x - T2 (a)	CCGACCGGAGGCGGGGTGGAGGGGGTCGAATTC

Supplementary Table 4

	Stop Codon			
	TGA	TAG	TAA	Total
**Negative Selection**				
Increased	19	8	34	61
Decreased	1	11	0	12
Total	20	19	34	73
**Positive Selection**				
Increased	12	3	3	18
Decreased	22	116	112	250
Total	34	119	115	268

Supplementary Table 4: Stop codons with significantly altered frequencies following selection. Nonsense mutations with significantly different frequencies after selection as determined by Fisher’s exact test are separated by codon, selection, and direction of change.
